# Aging‐induced Alternation in the Gut Microbiota Impairs Host Antibacterial Defense

**DOI:** 10.1002/advs.202411008

**Published:** 2025-01-10

**Authors:** Peng Gu, Rongjuan Wei, Ruofan Liu, Qin Yang, Yuxuan He, Jianbin Guan, Wenhao He, Jiaxin Li, Yunfei Zhao, Li Xie, Jie He, Qingling Guo, Jiajia Hu, Jingna Bao, Wandang Wang, Jiayin Guo, Zhenhua Zeng, Zhongqing Chen, Yong Jiang, Zhanguo Liu, Peng Chen

**Affiliations:** ^1^ Department of Critical Care Medicine Zhujiang Hospital Southern Medical University Guangzhou 510280 China; ^2^ Department of Pathophysiology Guangdong Provincial Key Laboratory of Proteomics School of Basic Medical Sciences Southern Medical University Guangzhou 510515 China; ^3^ Department of Gastroenterology The Seventh Affiliated Hospital of Southern Medical University Foshan 528244 China; ^4^ Department of Critical Care Medicine Nanfang Hospital Southern Medical University Guangzhou 510510 China; ^5^ Department of Clinical Medicine Laboratory Affiliated Xiaolan Hospital Southern Medical University Zhongshan 528415 China; ^6^ NMPA Key Laboratory for Research and Evaluation of Drug Metabolism Guangdong Provincial Key Laboratory of New Drug Screening School of Pharmaceutical Sciences Southern Medical University Guangzhou 510515 China; ^7^ Department of Respiratory and Critical Care Medicine The Tenth Affiliated Hospital Southern Medical University Dongguan 523059 China

**Keywords:** aging, bacterial infection, gut microbiome, macrophage

## Abstract

Older individuals experience increased susceptibility and mortality to bacterial infections, but the underlying etiology remains unclear. Herein, it is shown that aging‐associated reduction of commensal *Parabacteroides goldsteinii* (*P. goldsteinii*) in both aged mice and humans critically contributes to worse outcomes of bacterial infection. The colonization of live *P. goldsteinii* conferred protection against aging‐associated bacterial infections. Metabolomic profiling reveals a protective compound, apigenin, generated by *P. goldsteinii*, antagonizes bacterial clearance defects in aged mice. AMP‐binding protein (*ampB*) is identified as a key gene involved in apigenin synthesis in *P. goldsteinii* using homologous recombination in bacteria. Mechanistically, apigenin binds directly to the potential sites on Fgr (M341 and D404), preventing its inhibitory role on Vav1 phosphorylation, and therefore promoting the activation of Cdc42/Rac1, Arp2/3 expression and subsequent actin reorganization, which contributes to the enhanced phagocytosis of macrophages to bacteria. Collectively, the findings suggest that dysbiosis of the gut microbiota may impair host defense mechanisms and increase susceptibility to bacterial infections in older adults and highlight the microbiota‐apigenin‐Fgr axis as a possible route to ameliorate aging‐associated antibacterial defects.

## Graphical abstract

Bacterial infections in older individuals often result in worse outcomes, but the mechanisms remain unclear. This study reveals that aging‐induced alterations in gut microbiota impair host antibacterial defense mechanisms, and exacerbate the consequences of bacterial infections. These findings may offer valuable insights for the individualized treatment of bacterial infections in older adults.

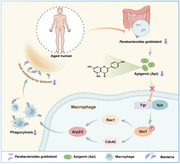

## 1 Introduction

The increasing prevalence and severity of bacterial infection is becoming an urgent public health threat affecting vulnerable populations, such as older hospitalized patients,^[^ ^1^ ^]^ particularly in intensive care units (ICU), and accounts for one‐third of deaths in this population globally.^[^ ^2^, ^3^ ^]^ Furthermore, with the aging of the population, the incidence and mortality of bacterial infection in hospitals increase exponentially in people ≥65 years of age.^[^ ^4^, ^5^ ^]^ Despite advancements in antibiotic therapy, infectious diseases remain a significant cause of mortality among older adults, and the emergence of antimicrobial‐resistant pathogens can lead to devastating diseases and significant costs to the global economy.^[^ ^6^, ^7^ ^]^ Therefore, further investigation of bacterial pathogens in older adults is necessary, and the design of novel treatment strategies to enhance host responses is needed to achieve the timely clearance of bacteria and improve prognosis.

Abundant literature suggests that the gut microbiome may offer defense against bacterial infections through the direct suppression of pathogens or through indirect effects on the host's functions.^[^ ^8^, ^9^, ^10^, ^11^ ^]^ Specifically, gut microbiota and metabolites, such as D‐lactate and phenylpyruvate, have excellent properties in promoting bacterial clearance by macrophages,^[^ ^11^, ^12^ ^]^ providing strong evidence supporting further investigation of gut microbial metabolites with enhanced bacterial clearance in older patients as potential therapeutic options. In this study, we systematically investigated the impact of aging‐induced alterations in the gut microbiota and metabolites on the susceptibility and severity of bacterial infection in aged hosts and found that the microbial‐derived metabolite, apigenin (Api), was decreased in both aged mice and humans. These findings could be valuable in understanding the mechanistic heterogeneity of aging‐associated bacterial infections and limiting bacterial infections in older adults.

## 2 Results

### 2.1 Aging Worsened Bacterial Infection Outcomes in a Gut Microbiota‐Dependent Manner

**Figure 1:**
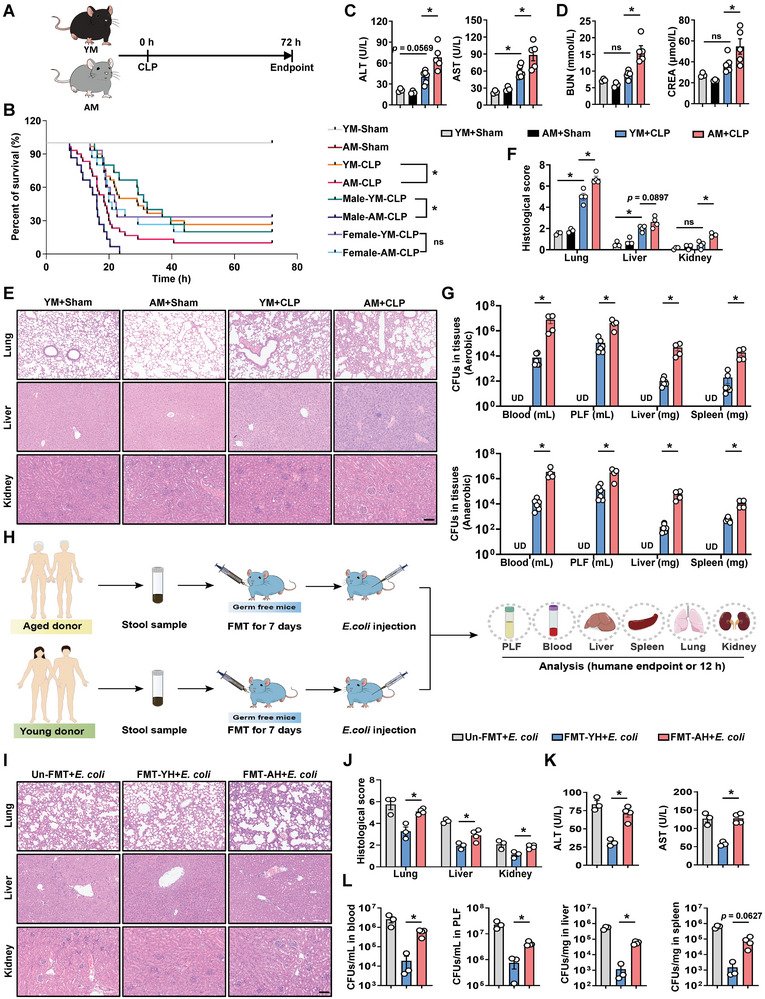
Influence of gut microbiota on the susceptibility to aging‐associated bacterial infection. A) Schematic illustration of the experimental design. B) Kaplan–Meier survival curves of YM and AM (*n* = 6 for YM‐sham and AM‐sham; *n* = 15 for Male‐YM‐CLP, Male‐AM‐CLP, Female‐YM‐CLP, and Female‐AM‐CLP; *n* = 30 for YM‐CLP and AM‐CLP). C) YM and AM underwent CLP or sham surgery. Samples were collected after euthanasia at humane endpoint or 12 h after surgery. Plasma ALT and AST levels. *n*  =  3–6. D) Plasma BUN and CREA levels. n  =  3–6. E) Representative H&E staining of lungs, liver, and kidneys sections. Scale bar, 100 µm. F) Pathological scores of H&E staining. n  =  3–4. G) Bacterial load in blood, peritoneal lavage fluid (PLF), liver, and spleen under aerobic or anaerobic culture conditions. n  =  3–6. H) GF mice received fecal microbiota transplantation (FMT) from young or aged human donors for 7 days before undergoing intraperitoneal injection of *E. coli*. Mice were euthanized at humane endpoint or 12 h after *E. coli* injection. I,J) H&E staining and pathological scores of the lungs, liver, and kidneys. n  =  3–4. Scale bar, 100 µm. K) Plasma concentrations of ALT and AST. n  =  3–4. L) *E. coli* colonies in blood, PLF, liver, and spleen. n  =  3–4. Data are presented as mean ± s.e.m. The Kaplan–Meier method with the log‐rank test. Statistical analysis was performed using One‐way ANOVA with Sidak's multiple comparison or two‐tailed Student's *t*‐tests (J–L). *p < 0.05. ns, not significant. UD, undetected.

To compare susceptibility to bacterial infections between aged mice (AM, 20‐month‐old) and young mice (YM, 3‐month‐old), cecal ligation and puncture (CLP) was initially used to establish the polymicrobial infection model and reflect the bacterial infection outcomes in YM and AM (**Figure** **1**A). The survival rates 72 h after CLP in AM and YM were 10% and 26.67% (*p* = 0.0041), respectively, with AM dying earlier and more pronounced differences in male mice (Figure 1B). We further investigated the impact of aging on the response to bacterial infection in male mice. Abdominal bacterial infections are associated with multiple organ damage (MOD). As expected, AM showed worsening MOD, including elevated plasma levels of alanine aminotransferase (ALT), aspartate aminotransferase (AST, Figure 1C), blood urea nitrogen (BUN), and creatinine (CREA, Figure 1D), as well as histological changes in multiple tissues, as indicated by hematoxylin and eosin (H&E) staining (Figure 1E,F). In addition, AM showed a significantly increased number of bacterial colony‐forming units (CFUs) in the blood, peritoneal lavage fluid (PLF), liver, and spleen compared to YM (Figure 1G). To further illustrate the attributable role of infectious insult in this phenotype, mice were subjected to intraperitoneal injection of *Escherichia coli* strain (*E. coli*, ATCC 25922) (Figure S1A, Supporting Information). Consistent with the above findings, AM showed an exacerbated organ damage and bacterial load during bacterial infection (Figure S1B–H, Supporting Information). Taken together, these results indicate that aging may increase the severity of MOD induced by bacterial infection as well as an insufficient capacity for bacterial clearance.

To determine whether the intestinal microbiome affected AM susceptibility to bacterial infections, AMs were pretreated with a cocktail consisting of four antibiotics for 5 days by oral gavage and then received fecal microbiome transplantation (FMT) from YM or AM (Figure S2A, Supporting Information). Following intraperitoneal injection of *E. coli*, the levels of ALT, AST, BUN, and CREA were also increased in AM received fecal microbial suspensions from AM (FMT‐AM+*E. coli*) as compared to those received fecal microbial suspensions from YM (FMT‐YM+*E. coli*) (Figure S2B–E, Supporting Information). Histopathological examination also revealed that AM, which received fecal microbial suspensions from AM (FMT‐AM+*E. coli*), showed significant worsening of MOD induced by bacterial infection (Figure S2F,G, Supporting Information). Moreover, bacterial load analysis showed that AM that received fecal microbial suspensions from AM (FMT‐AM+*E. coli*) had more live bacteria in the blood, PLF, liver, and spleen tissues than those that received feces from YM (FMT‐YM+*E. coli*, Figure S2H, Supporting Information). Next, we evaluated the contribution of the microbiota in aged humans (AH) to susceptibility to bacterial infection by FMT (Figure 1H; Figure S2I, Supporting Information). Stool samples from AH and young humans (YH) were collected and gavaged to antibiotic‐treated AM and germ‐free mice (GF). As speculated, mice received fecal microbial suspensions from AH (FMT‐AH+*E. coli*) revealed a significant aggravation of bacterial infection‐induced MOD (Figure S2J–O, Supporting Information) and bacterial burden in multiple tissues (Figure S2P, Supporting Information) when compared with mice transplanted with YH fecal microbial suspensions (FMT‐YH+*E. coli*). More importantly, GF mice transplanted with fecal microbial suspensions of AH showed significantly more serious MOD induced by bacterial infection in the lungs, liver, and kidneys, as indicated by H&E staining, as well as significantly increased plasma ALT and AST levels (Figure 1I–K). Consistent with histological and biochemical examinations, the bacterial load in multiple tissues, including the blood, PLF, liver, and spleen, was significantly increased in GF mice transplanted with fecal microbial suspensions of AH (Figure 1L). Collectively, these findings indicate that the altered microbiota induced by aging is at least partially responsible for worse outcomes in AM after bacterial infection.

### 2.2 Aging Induced Alteration of the Gut Microbiome and Reduction of *P. goldsteinii* in Both AM and AH

**Figure 2:**
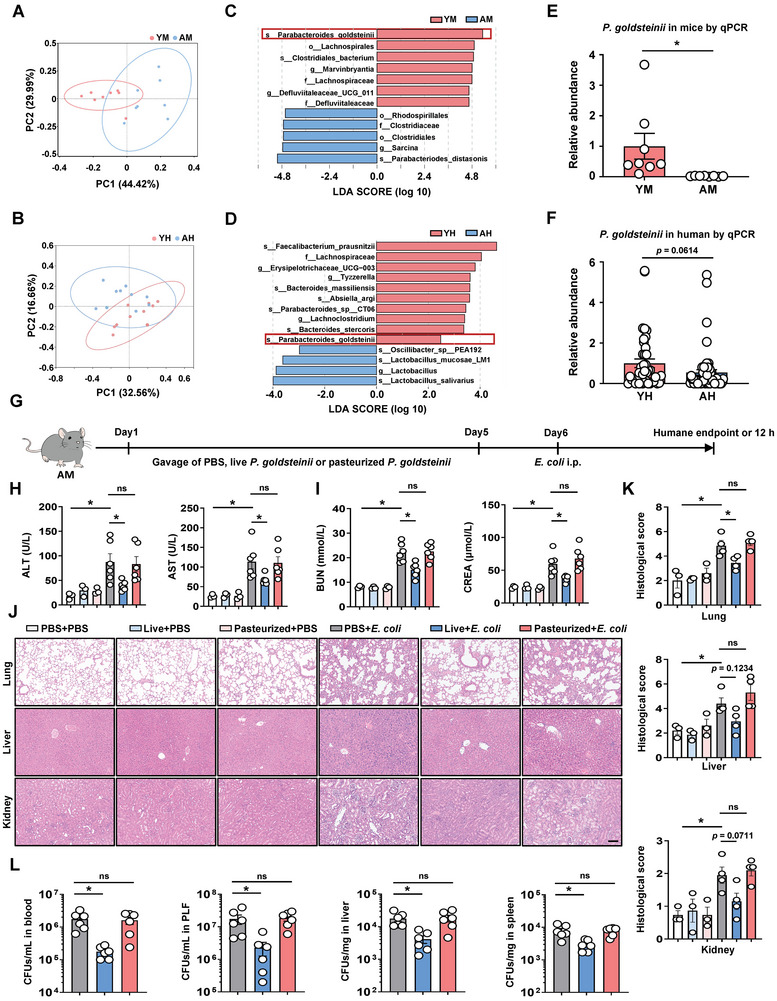
Identification of commensal microbiota difference and protective effect of live *P. goldsteinii* on bacterial infection in AM. A,B) Principal component ordination analysis of gut microbiota between aged and young mice (A, *n* = 8) or humans (B, *n* = 10) using the weighted Unifrac distance. Each point represents a sample, the colors represent different groups, and the ellipse represents 95% confidence interval. C, D) Linear discriminant analysis of differences in fecal microbiota profile in aged and young mice (C) or humans (D). Red color indicates enriched taxa in the young group, and blue color indicates enriched taxa in the aged group. For mice. *n* = 8; for humans, *n* = 10. E,F) The relative abundance of *P. goldsteinii* between aged and young individuals was validated by quantitative PCR. (n = 8 for mice, *n* = 40–56 for humans). G) Schematic overview of the treatment of *P. goldsteinii* in AM. AM were orally gavaged with PBS, live *P. goldsteinii* or pasteurized *P. goldsteinii* for 5 days followed by intraperitoneal injection of *E. coli* at day 6. 12 h after *E. coli* injection or at humane endpoint, mice were euthanized to detect bacterial load and multiple organ injury. H) Plasma levels of ALT and AST. *n* = 3–6. I) Plasma levels of BUN and CREA. *n* = 3–6. J) H&E staining for pathologic diagnosis of mice lungs, liver, and kidneys. Scale bar: 100 µm. K) Pathological scores of H&E staining. n  =  3‐4. L) Quantified results of bacterial load in blood, PLF, liver, and spleen samples. *n* = 6. Data are presented as mean ± s.e.m. Statistical analysis was performed using One‐way ANOVA with Sidak's multiple comparison, E) two‐tailed Student's *t*‐tests, or F) Mann–Whitney U test. *p < 0.05. ns, not significant.

To explore the potential involvement of gut microbiota in mediating aging‐induced susceptibility to bacterial infection, we initially performed 16S rDNA sequencing of stool samples from young and aged groups (including mice and humans). Although we did not find significant differences in alpha diversity based on Simpson analysis (Figure S3A,B, Supporting Information), intestinal microbiota compositional discrimination was observed by weighted Unifrac (**Figure** **2**A,B) and Bray‐Curtis principal component analysis (PCA, Figure S3C,D, Supporting Information) between the young and old groups. Furthermore, aging induced a distinct signature in the composition of the gut microbiome community at the phylum level, in both humans and mice (Figure S3E,F, Supporting Information). The heatmap determined by differential abundance analysis demonstrated that some species, including *Parabacteroides goldsteinii* (*P. goldsteinii*), *Clostridium aldenense*, *Bacteroides caecimuris*, and *Burkholderiales bacterium*, were significantly decreased in AM (Figure S3G, Supporting Information). Among these, *P. goldsteinii* showed the most pronounced difference, as indicated by linear discriminant analysis (LDA, Figure 2C). Although *P. goldsteinii* did not decrease the most among the reduced species (*Fecalibacterium prausnitzii*, *Bacteroides massiliensis*, *Absiella argi*, *Parabacteroides sp CT06*, *Bacteroides stercoris*, and *P. goldsteinii*) in AH fecal samples (Figure 2D; Figure S3H, Supporting Information), it was the only species that showed a decrease in both AM and AH in the current experimental setting (Figure 2C–F), suggesting the potential role of *P. goldsteinii* in aging‐associated phenotype alteration.

### 2.3 Live *P. goldsteinii* Ameliorated Bacterial Infection in AM

To investigate the effects of *P. goldsteinii* on bacterial infection in AM, we first gavaged AM with PBS, live *P. goldsteinii* (1 × 10^8^ CFUs per mouse), and pasteurized *P. goldsteinii* (1 × 10^8^ CFUs per mouse) for 5 days followed by intraperitoneal injection of *E. coli* on day 6 (Figure 2G). Next, we found that the liver and kidney functional parameters (ALT, AST, BUN, and CREA) were decreased in the plasma from AM subjected to intragastric pretreatment with live *P. goldsteinii* compared to PBS‐treated AM (Figure 2H,I). Consistently, the severe MOD, including the lungs, liver, and kidneys, observed in AM treated with PBS, was also significantly improved in AM with live *P. goldsteinii* pretreatment (Figure 2J,K). Furthermore, pretreatment with live *P. goldsteinii* resulted in a substantial reduction in the overall bacterial burden in AM samples (blood, PLF, liver, and spleen). Figure 2L). However, no significant difference was observed in MOD and bacterial load from PBS‐ or pasteurized *P. goldsteinii*‐pretreated AM (Figure 2H–L), implying that the beneficial effect of *P. goldsteinii* against AM bacterial infection may arise from the generation of specific protective metabolites.

### 2.4 Aging Individuals Showed Decreased Apigenin in Gut Metabolites

**Figure 3:**
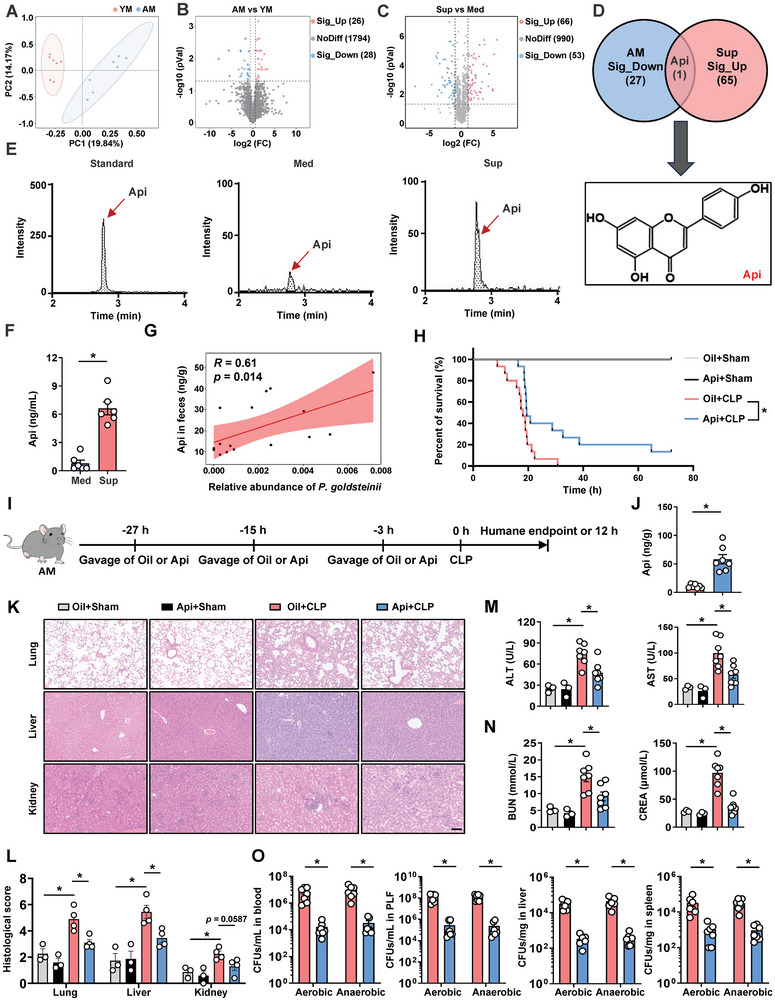
*P. goldsteinii* generation of apigenin (Api) alleviates bacterial infection in AM. A) Score plots of principal component analysis (PCA) of stool metabolites between YM and AM. *n* = 7. B) The outliers of metabolites are indicated in the volcano plot of stool metabolomics of YM and AM. *n* = 7. C) Volcano plot for differential metabolites in comparison between blank medium and cultural supernatant of *P. goldsteinii*. *n* = 4. D) Venn diagram indicating the joint compounds between the decreased compounds in stool of AM and enriched compounds in cultural supernatant of *P. goldsteinii*. E,F) LC‐MS/MS profiles showing the representative chromatograms (E) and quantitative concentration (F) of Api in the blank medium and *P. goldsteinii* culture supernatant. *n* = 6. G) Linear association between Api concentration and relative abundance of *P. goldsteinii* in mice by linear model. Correlations were assessed by nonparametric Spearman's test. *n* = 16. H) Kaplan–Meier survival curves of AM treated with Api or equal volume oil. *n* = 3–15. I) Experimental design involved the administration of Api to AM prior to CLP surgery. Mice were euthanized at the humane endpoint or 12 h after CLP operation. J) The Api concentration in fecal samples of AM gavaged with oil or Api. *n* = 7. K) Representative H&E‐stained histologic images of lung, liver, and kidney tissues from oil or Api‐treated AM. Scale bar: 100 µm. L) Pathological scores of H&E staining. n  =  3‐4. M) Plasma ALT and AST levels. *n* = 3–7. N) Plasma BUN and CREA levels. *n* = 3–7. O) The bacterial burdens in blood, PLF, liver, and spleen. *n* = 7. Data are presented as mean ± s.e.m. The Kaplan–Meier method with the log‐rank test. Statistical analysis was performed using One‐way ANOVA with Sidak's multiple comparison or two‐tailed Student's *t*‐tests. *p < 0.05.

To reveal functional metabolites that may improve aging‐associated bacterial infection, we determined changes in fecal metabolites between YM and AM by non‐targeted metabolomic analysis of mouse stool. The utilization of PCA showcased a distinct separation of metabolites derived from AM fecal samples compared to those from the YM group (**Figure** **3**A). This observation was further corroborated by partial least squares discriminant analysis (PLS‐DA, Figure S4A, Supporting Information). Differential metabolites were identified in AM as compared with YM, and 28 metabolites were significantly decreased in the stool of AM (Figure 3B; Figure S4B, Supporting Information). Next, we performed non‐targeted metabolomic analysis of the blank medium and culture supernatant of *P. goldsteinii* to identify potential protective substances secreted by live *P. goldsteinii*. PLS‐DA analysis revealed that the metabolite profiles of *P. goldsteinii* supernatants were significantly different from those of blank medium (Figure S4C, Supporting Information). Approximately 66 compounds showed a notable increase in the culture supernatant of *P. goldsteinii*, as indicated by the volcano plot (Figure 3C). Interestingly, Api experienced a significant decline in AM feces, whereas it was abundantly present in *P. goldsteinii* culture supernatants (Figure 3D; Figure S4D, Supporting Information). Furthermore, we verified the content of Api in the culture supernatant of *P. goldsteinii* and fecal samples of mice and humans by liquid chromatography/tandem mass spectrometry analysis (LC‐MS/MS), and a similar trend was observed (Figure 3E,F; Figure S4E, Supporting Information). Additionally, AM and AH had significantly lower Api levels in the plasma samples in the current experimental setting (Figure S4F, Supporting Information). We further confirmed that Api concentration in AM stools was positively correlated with *P. goldsteinii* abundance (Figure 3G). A previous study showed that Api, a flavonoid compound, can be obtained through food consumption.^[^ ^13^ ^]^ Therefore, we designed a flavonoid‐free diet to eliminate the possible impact of dietary variation on the decline of Api in the aged groups. The Api content in plasma and feces remained lower in AM fed a flavonoid‐free diet than in YM (Figure S4G, Supporting Information), suggesting a lower concentration of Api in the aged group could be associated with the decreased abundance of *P. goldsteinii*, and may lead to increased susceptibility to bacterial infection in the aged group.

### 2.5 Apigenin Improved Bacterial Clearance and Protected Against Bacterial Infection in AM

To explore the potential functional roles of Api in the progression of bacterial infection, AM was orally administered (10 mg kg^−1^) three times at ‐27, ‐15, and ‐3 h and then subjected to CLP surgery. A survival study showed that Api administration significantly improved survival outcomes in AM, with a lower mortality rate than in the control group (Figure 3H,I). Administration of Api also significantly reversed the decrease in fecal Api concentration in Api‐treated AM compared to that in oil‐treated AM (Figure 3J). Furthermore, MOD in AM subjected to CLP or *E. coli* injection (involving functional parameters of the liver and kidney: ALT, AST, BUN, and CREA, as well as pathological changes in the lungs, liver, and kidneys) was also markedly improved by Api pretreatment (Figure 3K–N; Figure S5A–G, Supporting Information), and a significantly reduced bacterial load in blood, PLF, liver, and spleen samples was also observed in the Api treatment group (Figure 3O; Figure S5H, Supporting Information).

**Figure 4:**
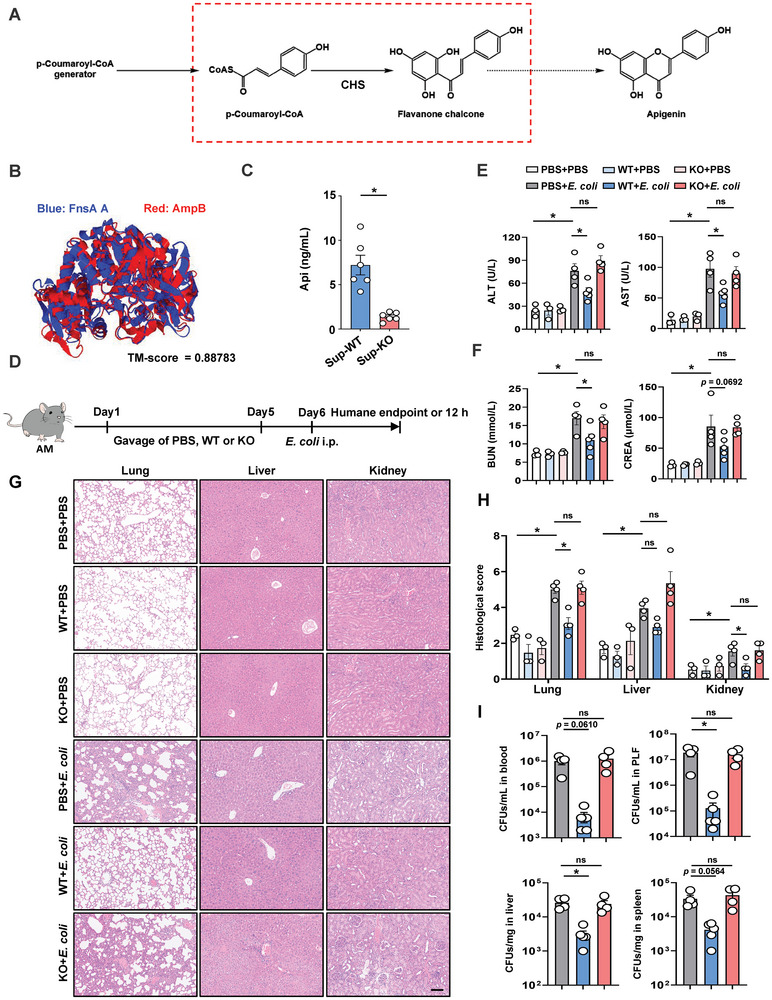
Deletion of *ampB* impairs the protective effect of *P. goldsteinii* on bacterial infection. A) Schematic illustration of Api generation. The key step and enzyme are highlighted. CHS: chalcone synthase. B) Structural comparison using the US‐align. FnsA: fungal naringenin synthase. C) Api concentration in the culture supernatant of WT or *ampB*‐KO *P. goldsteinii*. n  =  6. D) Schematic overview of experimental design for studying the impact of WT or *ampB*‐KO *P. goldsteinii* on bacterial infection in AM. Mice were euthanized at the humane endpoint or 12 h after *E. coli* injection. E) Plasma concentrations of ALT and AST. *n* = 3–5. F) Plasma concentrations of BUN and CREA. *n* = 3–5. G) Representative H&E staining images of lungs, liver, and kidneys in WT‐ or *ampB*‐KO *P. goldsteinii*‐treated AM. Scale bar: 100 µm. H) Pathological scores of H&E staining. n  =  3–4. I) Bacterial burdens in blood, PLF, liver, and spleen after infection. *n* = 4–5. Data are presented as mean ± s.e.m. Statistical analysis was performed using One‐way ANOVA with Sidak's multiple comparison or two‐tailed Student's *t*‐tests. *p < 0.05. ns, not significant.

The formation of Api, a key flavonoid, has been extensively studied in plants, fungi, and bacteria,^[^ ^14^, ^15^, ^16^ ^]^ but not in *P. goldsteinii*. Generally, the starter unit for Api biosynthesis is phenylalanine or L‐tyrosine, which is converted to Api after four or five enzyme‐catalyzed reaction steps. To the best of our knowledge, although the biosynthetic modes of Api are diverse, chalcone synthase is necessary for all modes (**Figure** **4**A). Therefore, we screened for the potential biosynthetic genes responsible for the generation of Api in *P. goldsteinii*. Amidst the potential chalcone‐synthetic proteins in *P. goldsteinii*, *ampB* (AMP‐binding protein) shared some similarities (TM score = 0.88783) with the catalytic domain of the recognized chalcone synthase through the employment of Universal Structural alignment^[^ ^14^, ^17^ ^]^ (US‐align, Figure 4B), which is essential for substrate recognition and activation. To further illustrate the mechanism of Api production by *P. goldsteinii*, the *ampB* gene was knocked out in *P. goldsteinii* and verified by PCR (Figure S6A,B, Supporting Information). Compared to wild‐type (WT) *P. goldsteinii*, the deletion of *ampB* in *P. goldsteinii* caused a loss of its ability to generate Api, although no significant difference in the growth curve was observed between WT and *ampB*‐KO *P. goldsteinii* (Figure 4C; Figure S6C, Supporting Information). Next, we inoculated AM with PBS, WT, or *ampB*‐KO *P. goldsteinii* (Figure 4D). The deletion of *ampB* did not influence the colonization ability of *P. goldsteinii* (Figure S6D, Supporting Information). However, *ampB*‐KO *P. goldsteinii* lost the beneficial effects against MOD induced by bacterial infection (Figure 4E–H), and the bacterial load was significantly higher in AM treated with *ampB‐*KO *P. goldsteinii* than in those treated with WT *P. goldsteinii* (Figure 4I). Furthermore, we pretreated GF mice with live WT or *ampB*‐KO *P. goldsteinii* for five days (Figure S6E, Supporting Information) and found that GF mice treated with *ampB*‐KO *P. goldsteinii* also showed an increased MOD (Figure S6F,G, Supporting Information), plasma ALT and AST levels (Figure S6H,I, Supporting Information), and had a greater number of bacterial CFUs in the blood, PLF, liver, and spleen samples (Figure S6J, Supporting Information). Collectively, our data revealed that live *P. goldsteinii*‐derived Api could improve bacterial clearance and protect against bacterial infections in AM.

### 2.6 Apigenin Enhanced Macrophage Phagocytosis

**Figure 5:**
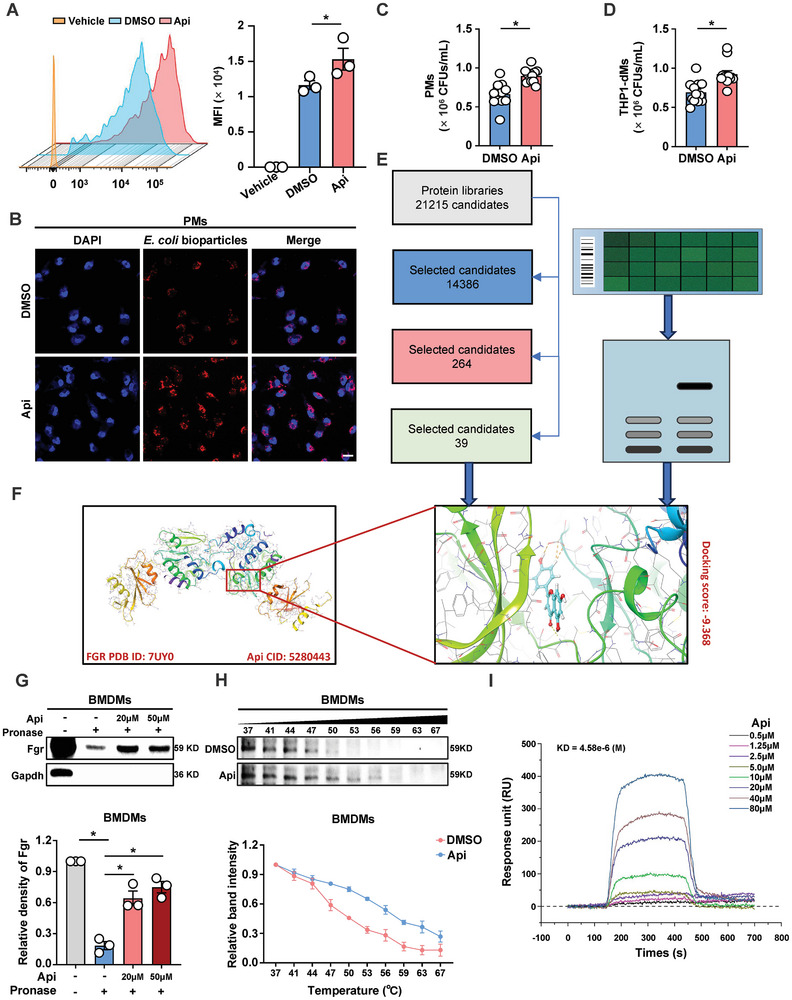
Api binds to Fgr and enhances macrophage phagocytosis. A) Flow cytometric analysis of in vivo peritoneal macrophage phagocytosis of pHrodo red *E. coli* BioParticles injected into AM previously treated with DMSO or Api. *n* = 3. B) Confocal micrographs show phagocytosis of pHrodo red *E. coli* bioparticles by peritoneal macrophages (PMs) collected after 3 h of Api treatment. Scale bar: 10 µm. C,D) Measurement of phagocytic function of DMSO‐ or Api‐treated PMs and THP1‐dMs using *E. coli* stimulation for 45 minutes. *n* = 12. E) Virtual screening approach to capture the potential targets of Api. F) A model of Api binding with Fgr protein generated by molecular docking. G) BMDMs lysates were treated with DMSO or different concentrations of Api, and then incubated with pronase. The expression of Fgr was detected by immunoblotting. *n* = 3 independent experiments. H) CETSA assay was used to evaluate the binding between Api and Fgr in thermodynamic levels. The representative image of Fgr immunoblotting and quantitative results were presented. *n* = 3 independent experiments. I) Interaction between Api and recombinant Fgr protein was analyzed by SPR assay. Data are presented as mean ± s.e.m. Statistical analysis was performed using One‐way ANOVA with Sidak's multiple comparison or two‐tailed Student's *t*‐tests. *p < 0.05.

To assess the impact of Api on bacterial growth, *E. coli* was co‐cultured with varying concentrations of Api. The presence of Api did not lead to any noticeable variance in bacterial growth when compared to the control group (Figure S7A, Supporting Information), suggesting that Api might influence the eradication of bacteria by enhancing the bactericidal efficacy of host immune cells. Macrophages, as the front‐line host defense against microbial infections, recognize microbial pathogens and phagocytose foreign bodies to facilitate the clearance of pathogens in the host.^[^ ^18^, ^19^ ^]^ During bacterial infection, a potential explanation for the beneficial effect of Api is the enhanced control of the source in the aged group. To test this hypothesis, pHrodo red *E. coli* BioParticles were injected into the peritoneal cavity of AM treated with either Api or DMSO. One hour following the injection, the mice were euthanized, and the resident peritoneal macrophages were used to evaluate the phagocytic activity by monitoring changes in PE fluorescence. Mice treated with Api demonstrated significantly greater phagocytosis of peritoneal macrophages compared to the control group that received DMSO (**Figure** **5**A). Next, we conducted Cell Counting Kit‐8 (CCK‐8) assays to evaluate the cytotoxicity of Api at concentrations ranging from 1 to 50 µM in peritoneal macrophages (PMs), bone marrow‐derived macrophages (BMDMs), and THP1 derived macrophages (THP1‐dMs). No discernible detrimental effects on cell viability were observed at the tested concentrations (Figure S7B, Supporting Information), leading us to select 20 µM of Api for subsequent experiments. Consistent with our expectations, Api treatment significantly augmented the phagocytic activity of macrophages toward heterologous bodies, including pHrodo red *E. coli* BioParticles and *E. coli* (Figure 5B–D; Figure S7C, Supporting Information). In addition, more filopodia were formed around the membrane, and lamellipodial protrusions were observed in the Api‐treated macrophages (Figure S7D,E, Supporting Information). However, no significant differences were observed in Api‐ or DMSO‐treated neutrophils, another important phagocyte (Figure S7F, Supporting Information). Furthermore, we found that Api‐treated AM showed equivalent numbers of leukocytes to DMSO‐treated AM (Figure S7G, Supporting Information), indicating that supplementation with Api did not significantly affect leukocyte counts, but may increase the phagocytosis of macrophages.

### 2.7 Apigenin Bound Directly to Fgr and Promoted Macrophage Phagocytosis through the Vav1‐Rac1/Cdc42‐Arp2/3 Axis

To further identify the host‐responsive protein that mediates the increased phagocytosis of macrophages with Api treatment, a drug affinity‐responsive target stabilization (DARTS) assay was employed to identify the potential molecular target of Api (Figure S8A, Supporting Information). Coomassie blue staining revealed that Api enhanced the anti‐protease hydrolysis of a protein within the 55–70 kDa range (Figure S8B, Supporting Information). We focused on Fgr because 1) it was the protein with the highest ranking among proteins falling within the expected molecular weight range (55–70 kDa) after a virtual screen using the molecular docking/scoring strategy (Figure 5E,F), and 2) Fgr has been reported as a negative regulator of phagocytosis in murine macrophages.^[^ ^20^ ^]^ To confirm this hypothesis, immunoblot analysis of the DARTS sample was performed to reveal the increased stabilization of Fgr during proteolysis when BMDMs (Figure 5G) and THP1‐dMs (Figure S8C,D, Supporting Information) were treated with Api. We then performed a cellular thermal shift assay (CETSA) and demonstrated that Api could increase the thermal stability of Fgr in heat‐denatured BMDMs and THP1‐dMs (Figure 5H; Figure S8E,F, Supporting Information). A further surface‐plasmon resonance (SPR) analysis confirmed the binding activity between Api and Fgr in a dose‐dependent manner (Figure 5I).

**Figure 6:**
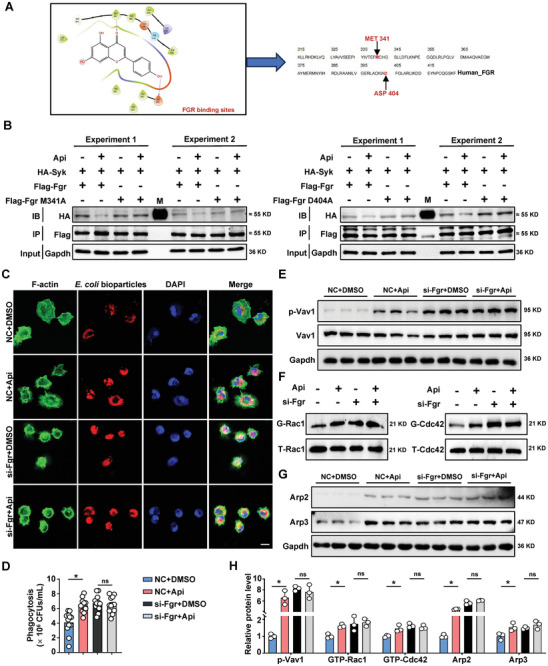
Api relies on Fgr to enhance phagocytosis of macrophages. A) The Fgr residues that are likely to participate in the interactions with Api are labeled. B) HA‐tagged Syk and two mutant forms of Flag‐tagged Fgr were co‐transferred to THP1‐dMs with or without Api treatment. IP with anti‐Flag antibody followed by western blotting to analyze the effects of Api treatment on the binding of Fgr with Syk. C) PMs were transfected with normal control (NC) and si‐Fgr with or without Api treatment. Confocal immunofluorescence images showing colocalization of pHrodo red *E. coli* bioparticles (red), F‐actin (green), and DAPI (blue). Scale bar: 10 µm. D) Phagocytosis of *E. coli* by PMs transfected with si‐Fgr or NC for 48 h in the presence or absence of Api. *n* = 12. E) Phosphorylated Vav1 expression in BMDMs transfected with NC or si‐Fgr, under DMSO or Api conditions. F) siRNA‐transfected BMDMs were treated with DMSO or Api and then analyzed for GTP‐bound forms of Rac1 or Cdc42. Lysates were used to assess total Rac1 or Cdc42. G) Arp2 and Arp3 expression in BMDMs transfected with NC or si‐Fgr, under DMSO or Api conditions. H) Quantification of p‐Vav1, GTP‐Rac1, GTP‐Cdc42, Arp2 and Arp3 expression. *n* = 3 independent experiments. Data are presented as mean ± s.e.m. Statistical analysis was performed using One‐way ANOVA with Sidak's multiple comparison. *p < 0.05. ns, not significant.

Binding of Fgr to Syk tyrosine kinase has been reported to prevent the association of Syk with Vav1.^[^ ^21^ ^]^ We confirmed that Fgr could bind to Syk, while Api treatment mitigated Fgr binding to Syk in macrophages, as shown by immunoprecipitation with FLAG‐tagged Fgr and monitoring HA‐tagged Syk (Figure S8G, Supporting Information). Moreover, Api promoted the phosphorylation of Vav1 (Figure S9A,B, Supporting Information) and upregulation of both GTP‐Rac1 and GTP‐Cdc42 (Figure S9C,D, Supporting Information), which in turn led to increased expression of Arp2/3 (Figure S9E,F, Supporting Information) during bacterial‐stimulated phagocytosis in both BMDMs and THP1‐dMs, indicating that Api can act as an Fgr inhibitor in the phagocytosis of macrophages in vitro. According to the molecular docking analysis between Api and the crystal structure of human Fgr, Api fit to the pocket of Fgr and interacted with two amino acid sites (M341 and D404, **Figure** **6**A). To further confirm whether the predicted sites contributed to the inhibition of Fgr by Api, THP1‐dMs were transferred with WT or site‐mutant Fgr plasmid (M341A or D404A) and HA‐tagged Syk with or without Api treatment. We found that mutation of M341A or D404A reversed the Api‐induced decrease in Fgr and Syk binding (Figure 6B), suggesting that Api may functionally bind to Fgr at the M341 and D404 sites.

To further validate the Fgr‐dependent effects of Api on macrophage phagocytosis, cells were transfected with siRNA‐Fgr and then treated with DMSO or Api. As expected, Fgr knockdown (relative to the negative control siRNA group) directly promoted macrophage phagocytosis, while significantly diminished the ability of Api to promote phagocytosis in macrophages (Figure 6C,D; Figure S10A, Supporting Information). Furthermore, the elimination of Fgr in macrophages by siRNA directly promoted the phosphorylation of Vav1, activation of Cdc42 and Rac1, and expression of Arp2/3, which could not be further enhanced by Api treatment (Figure 6E–H; Figure S10B, Supporting Information). Next, we transfected THP1‐dMs with an Fgr‐overexpressing (OE‐Fgr) plasmid (Figure S10C, Supporting Information). Phagocytic analysis using pHrodo red *E. coli* BioParticles or *E. coli* confirmed that Fgr overexpression reversed the promotion of macrophage phagocytosis by Api (Figure S10D,E, Supporting Information), and Api showed weak regulatory effects on the levels of phosphorylated Vav1, Cdc42/Rac1, and Arp2/3 proteins in Fgr‐overexpressing THP1‐dMs cells (Figure S10F–I, Supporting Information). In conclusion, these results suggest that the direct binding of Api and Fgr prevents the inhibitory role of Fgr in the phosphorylation of Vav1, activation of Cdc42 and Rac1, and expression of Arp2/3, thereby increasing macrophage phagocytosis.

## 3 Discussion

Infections rank as the primary cause of morbidity and mortality among the older populations.^[^ ^22^, ^23^ ^]^ Although various factors have been documented to influence the occurrence and intensity of infections in older individuals, including the presence of chronic comorbid diseases, declines in immune function, and alterations in normal physiological functions,^[^ ^24^, ^25^, ^26^ ^]^ it remains unclear why aging is associated with worse outcomes in bacterial infections. Previous studies have shown that aging impairs the effective myeloid response to bacterial infection, including reduced phagocytosis and chemotaxis.^[^ ^25^, ^27^ ^]^ However, to date, much research on susceptibility to bacterial infections in older adults has focused on the host's response to the pathogen, as opposed to the differences in the bacteria that originate in the gut microbiota. Here, we showed that aging‐driven alterations in the gut microbiota and metabolites may increase susceptibility to bacterial infection and worsen outcomes in the aged groups. First, we demonstrated that aging worsens bacterial infection outcomes in a gut microbiota‐dependent manner by transplanting the fecal microbiota of AM versus YM or AH versus YH donors into both antibiotic‐pretreated AM and GF mice. Notably, when we transplanted fecal microbial suspensions from AM into YM treated with antibiotics, the outcomes of bacterial infection in young mice did not worsen (data not shown), suggesting that YM may be more responsive to bacterial infections than AM, exhibiting enhanced phagocytosis, reactive oxygen species production, and chemotaxis. Additionally, previous studies have indicated that advancing age is accompanied by alterations in the gut microbiota.^[^ ^28^, ^29^, ^30^ ^]^ In particular, we found that the abundance of *P. goldsteinii* was decreased in both AM and AH, indicating that *P. goldsteinii* may be involved in susceptibility to bacterial infection in aged groups. Although *P. goldsteinii* has exhibited great potential to be a probiotic in anti‐inflammatory, anti‐obesity, and anti‐COPD agent,^[^ ^31^, ^32^, ^33^ ^]^ the interactions between *P. goldsteinii* and the host in the context of bacterial infection remain poorly understood. To this end, we further demonstrated an antagonistic relationship between live *P. goldsteinii* and the outcome of bacterial infection in male AM. It is noteworthy that previous reports indicate that biological sex may serve as a potential variable mediating the impact of aging on the abundance of *P. goldsteinii*.^[^ ^34^, ^35^ ^]^ We also found that aging‐related reduction of *P. goldsteinii* was more pronounced in the male population (data not shown). Therefore, it is necessary to further investigate whether supplementation with *P. goldsteinii* in aged female mice could also enhance resistance to bacterial infections. In addition, given the complexity of the gut environment, while the identification of *P. goldsteinii* supports the hypothesis that specific bacterial species can play a role in reducing bacterial infections in older populations, further investigation is needed to explore other potential gut candidates and the interactions among gut microbiota.

In addition to gut microbiota dysbiosis, decreased beneficial metabolites produced by commensal bacteria could aggravate bacterial infection in older adults. Gut microbiota‐related metabolites, such as vitamins, short‐chain fatty acids (SCFA), and amino acid catabolites, can reduce or exacerbate the susceptibility of the host to metabolic syndrome, tumors, and cardiovascular disease by agonizing and antagonizing their cognate receptors.^[^ ^36^, ^37^ ^]^ In the context of bacterial infection, supplementation with a gut microbiota‐derived peptide, amino acid metabolites, or SCFA can modulate the host's immune system and susceptibility to bacterial infection.^[^ ^12^, ^38^, ^39^ ^]^ Given that only live *P. goldsteinii* presents the beneficial effect on bacterial infection in AM, we therefore performed metabolomic analysis to uncover metabolites that could potentially mediate the ameliorating effect. We found that Api produced by *P. goldsteinii* was significantly reduced in AM and AH. Previous studies have reported that Api, a common flavonoid, exhibits a variety of biological activities, including antioxidant, antiviral, anti‐inflammatory, and anticancer properties in various cellular processes.^[^ ^40^, ^41^ ^]^ Our results expand the application of Api in improving bacterial infection in aged groups by antagonizing aging‐associated bacterial clearance defects. However, further studies are needed to determine whether apigenin can aid younger individuals in resisting bacterial infections. Notably, Api is a compound that can be synthesized by a variety of plants, bacteria, and fungi.^[^ ^14^, ^16^, ^40^ ^]^ Therefore, the sources of Api in the gut can be wide‐ranging, including obtained from food or synthesis by gut microbes. In order to better illustrate that *P. goldsteinii* is an important source of Api, we first found that the content of Api in the culture supernatant of *P. goldsteinii* was significantly higher than that in the blank culture. In addition, the feces and plasma from AM were still significantly lower than those from YM, after excluding the possible food source of Api. Therefore, we speculated that a decrease in the abundance of *P. goldsteinii* in AM may lead to a decrease in the concentration of Api. Additionally, we identified a key gene participated in Api production in *P. goldsteinii*. Recent studies have revealed the formation of Api in plants, fungi, and several other bacteria, and chalcone synthase is necessary for all different modes.^[^ ^42^, ^43^ ^]^ Here, we showed that the *ampB* gene in *P. goldsteinii* shares some similarities with the catalytic domain of a known chalcone synthase, suggesting that *ampB* is probably involved in Api biosynthesis in *P. goldsteinii*. We further confirmed the function of *ampB* by generating an *ampB*‐KO strain of *P. goldsteinii*, which produced negligible amounts of Api and lost its ability to prevent bacterial infection in AM and GF mice. These findings demonstrated how *P. goldsteinii* generates Api and provides a protective effect against bacterial infection in AM. Given the diversity and complexity of Api production in bacteria, we still cannot fully rule out the possibility that other bacteria or fungi in the gut can produce Api. Further research is needed to fully understand the mechanisms underlying gut microbial Api production.

As a crucial component of the innate immune system, macrophages are integral to the host's immune responses against microbial infections, primarily through phagocytosis and the subsequent degradation of pathogens. The phagocytosis process encompasses several critical stages, including the examination of bacteria, the formation of phagocytic cup, and the sealing of phagosome, all of which necessitate the remodeling of the actin cytoskeleton.^[^ ^19^ ^]^ The confocal imaging showed that Api treatment significantly promoted the formation of filopodia and lamellipodial protrusions around the membrane and improved the phagocytosis of heterologous bodies by AM macrophages. Another major finding of the present study was identifying that Api may bind directly to Fgr at the M341 and D404 sites. A recent study showed that Fgr kinase, a member of the Src kinase family, is required for the activation of proinflammatory macrophages during diet‐induced obesity.^[^ ^44^ ^]^ Furthermore, Fgr has previously been reported to bind to Syk kinase and negatively regulate phagocytosis in murine macrophages.^[^ ^20^, ^21^ ^]^ Here, we found that Api treatment could mitigate the binding of Fgr to Syk and promote the phosphorylation of Vav1. The small GTPases Cdc42/Rac1 are well‐known as critical regulators of actin cytoskeletal rearrangements that are necessary for phagocytosis.^[^ ^45^, ^46^ ^]^ Upon analysis of the signaling pathways, we found that Api treatment promoted the activation of Cdc42/Rac1 in response to bacterial stimulation. Moreover, Api‐treated macrophages exhibited upregulated expression of the Arp2/3 complex, an actin nucleator, and a downstream effector of Rac. These findings indicated that Api can potentially improve phagocytosis by directly interacting with Fgr, thereby preventing its inhibitory effect on Vav1 phosphorylation. Additionally, Api promoted the activation of Cdc42/Rac1, expression of Arp2/3, and subsequent actin reorganization in bacteria‐infected AM macrophages. Consequently, inhibitors like Api that target Fgr may serve as potential therapeutic agents for preventing bacterial infections in older adults.

Collectively, the present study provides an insight that aging‐induced impairment of antibacterial defense may be driven by the effects of aging on gut microbiota dysbiosis. The microbially‐derived metabolite, Api, could bind directly to Fgr at the potential sites M341 and D404, which in turn up‐regulates the phosphorylation of Vav1, activates of Cdc42 and Rac1, and enhances the expression of Arp2/3 to improve macrophage phagocytosis. The major implications of these findings: 1. better understanding the mechanistic heterogeneity of aging‐associated bacterial infection; 2. potentially guide the individualized treatment of bacterial infections in older adults.

## 4 Experimental Section

### Animals

All animal experiments in this study were carried out in compliance with the guidelines of the respective facilities. The Institutional Animal Care and Use Committee (IACUC) of Southern Medical University approved all protocols related to the use, treatment, and euthanasia of animals (permit: SMUL2022308). C57BL/6J mice, categorized as young (3‐month‐old) and aged (20‐month‐old), were obtained from Charles River Laboratory (Beijing, China). These mice represent the equivalent of young adult humans (∼20 years old) and aged humans (∼65 years old). Standard chow and autoclaved water were provided ad libitum. GF C57BL/6J mice (3‐month‐old) were purchased from Gnotobio Biotechnology (Shenzhen, China), maintained in sterile isolators, and provided autoclaved food and water. Animals were randomly allocated into each treatment group.

### Human Subjects

The Ethical Committee of Nanfang Hospital, Southern Medical University, approved the collection of fecal and plasma samples from the volunteers (NFEC‐2023‐320). Individuals from two specific age groups [aged from 20 to 40 years old (n = 40) or ≥ 65 years old (n = 56)] were recruited. The certain exclusion criteria for this cohort included: being pregnant or breastfeeding; currently having an active infection within the past two weeks; being diagnosed with inflammatory bowel disease, pulmonary tuberculosis, or AIDS; having any forms of cancer and alcoholism; having taken laxatives, antibiotics (including rifaximin), prebiotics, or probiotics within the past three months and being deemed unsuitable for biopsy by medical professionals. Fresh fecal and plasma samples were collected from the donors and promptly frozen at ‐80 °C until they were ready for use. The characteristics of the enrolled volunteers are presented in Tables S1 and S2 (Supporting Information).

### 16S rDNA Sequencing

Sterile tubes were used to collect fresh fecal pellets in an aseptic manner. Following the manufacturer's instructions, DNA extraction was carried out on the fecal samples from mice and humans using a SPINeasy DNA Kit for Feces (MP Biomedicals, 116531050). PCR amplification was performed using primers 515F (GTGCCAGCMGCCGCGGTAA)/806R (GGACTACHVGGGTWTCTAAT) with the ABI GeneAmp 9700 PCR thermocycler (ABI). Subsequently, the amplicons were sequenced on an Illumina PE300 platform (Illumina, San Diego, USA). The raw data generated by sequencing is stored in the FASTQ format, which includes identification information and sequencing quality scores for each sample. Divisive Amplicon Denoising Algorithm 2 (DADA2) within QIIME2 (version 2023.5.1) was used to trim low‐quality reads, eliminate chimeras, and identify amplicon sequence variants (ASVs).^[^ ^47^, ^48^ ^]^ For species annotation, the denoised OTU table was utilized as input and the greengenes2 plug‐in was employed in Qiime2 to obtain taxonomic information for each OTU.^[^ ^49^ ^]^ Once the species annotation process was completed, diversity analysis was conducted using the QIIME2 diversity plug‐in. The principal coordinate analysis (PCoA), utilizing either Bray‐Curtis distance or weighted Unifrac distance, was employed to assess the similarity among microbial communities found in various samples. To further explore and identify the taxa that are significantly more abundant within distinct groups (from phylum to species), linear discriminant analysis (LDA) effect size (LEfSe) was employed.^[^ ^50^ ^]^

### Metabolomic Analysis

For fecal samples, 100 mg of feces are weighed and mixed with 500 µL of methanol. The mixture is then ground for 5 minutes and centrifuged for 15 minutes at 4 °C and 12000 × rpm. Following centrifugation, the supernatant was collected in a 96‐well plate. In the case of supernatant samples, a designated volume of 100 µL was carefully transferred for each individual sample to occupy a solitary well within a 96‐well sample collection plate. Subsequently, each sample was supplemented with a measured quantity of 300 µL of acetonitrile. Following a thorough mixing process for a duration of 3 minutes, the plate was subjected to centrifugation at 4680 × rpm for 15 minutes under controlled conditions of 4 °C. Finally, all of the resulting supernatant was meticulously transferred to a single well within the 96‐well sample collection plate. The nontargeted metabolomics was performed by using the UPLC‐ESI‐QTOF MS system (Waters). The chromatographic column, ACQUITY UPLC HSS T3 [100 mm (length) × 2.1 mm (id), 1.8 µm; Waters] was used in this study. For positive mode, the aqueous phase A consisted of 0.1% (v/v) formic acid in water, while the organic phase B contained 0.1% (v/v) formic acid in acetonitrile. For negative mode, solvent A was comprised of 10 mm ammonium formate in water, whereas solvent B was composed of acetonitrile. The separation process occurred in the following manner: 0% B–2% B from 0–0.2 minutes, 2% B–60% B from 0.2–4 minutes, 60% B– 60% B from 4–5 minutes, 60% B–95% B from 5–9 minutes; 95% B–95% B from 9–10.5 minutes, and finally holding at 2% B from 10.6–13 minutes with a flow rate of 0.4 mL min^−1^. The positive and negative spray voltages used were 3.0 kV and ‐2.5 kV, respectively. Progenesis QI (Waters) was employed as a tool for selecting and aligning peaks for the identification of differential metabolites during analysis.

### Transient Transfection

RiboBio (Guangzhou, China) synthesized the siRNA and NC oligonucleotide sequences. The Fgr‐overexpression plasmids and mutant Fgr plasmids were synthesized by GENEYUAN Bio‐Tec (Guangzhou, China). Lipofectamine 3000 (Invitrogen) was used to transform either Fgr siRNA or overexpression plasmid, following the guidelines provided by the manufacturer. Standard procedures were employed to perform quantitative PCR on cellular extracts after 48 h of transfection.

### Confocal Imaging

PMs or THP1‐dMs were pre‐treated as indicated in figure and then subjected to incubation with pHrodo red *E. coli* BioParticles (Thermo Fisher, P35361) for a duration of 30 minutes at 37 °C. If necessary, the cells were fixed in a solution of 4% paraformaldehyde in PBS for a span of 30 minutes, rinsed, and subsequently incubated with actin tracker green (Beyotime, C1033) at room temperature for a duration of 1 h. After three washes with PBS, the cell nuclei were stained using antifading mounting medium (Solarbio, S2110) with the inclusion of DAPI, followed by visualization using a confocal microscope (LSM 880, Carl Zeiss Microimaging, Jena).

### Drug Affinity‐Responsive Target Stability Assay

BMDMs and THP1‐dMs were lysed on ice for a duration of 30 minutes. After centrifugation (15 000 × *g*, 15 minutes), the lysates were diluted to achieve a consistent final volume and protein concentration with 1× TNC buffer. The diluted lysates were then combined with either Api at the designated concentration (20 or 50µM) or a DMSO control at room temperature for a duration of 2 h. Next, each sample underwent digestion with pronase (Roche, 10165921001) at a ratio of 1:300 (w/w) for a duration of 30 minutes.^[^ ^51^ ^]^ Subsequently, the lysates were supplemented with 5× loading buffer, boiled for 10 minutes, separated via SDS‒PAGE, and further analyzed through Coomassie Blue staining or western blotting analysis.

### Cellular Thermal Shift Assay

To conduct the cellular thermal shift assay (CETSA) experiments,^[^ ^52^ ^]^ the harvested cell suspensions underwent three cycles of freeze‐thaw using liquid nitrogen followed by centrifugation at 20000 × g for 20 minutes at 4 °C. Divide the supernatant collected after centrifugation into two aliquots: one treated with Api and the other treated with an equal volume of DMSO. Incubate these aliquots for two hours at room temperature. After treatment, each lysate was further divided into 10 aliquots and subjected to heating at different temperatures for 3 minutes each, followed by a 3‐minute cooling period at room temperature. To separate the cell debris, the lysates were centrifuged at 15 000 × rpm for 15 minutes at 4 °C. The resulting supernatants were boiled by adding 5 × loading buffer and then analyzed through western blotting.

### Surface‐Plasmon Resonance

The PlexArray HT surface plasmon resonance imaging platform (SPRi) was utilized to determine the binding affinity of Api to recombinant human FGR (rhFGR, Abnova, H00002268‐P01). To initiate the experiment, the rhFGR samples were dissolved in PBS and then immobilized on the chip. Activation was achieved by treating the chip with 10 mM NiSO_4_. Subsequently, various concentrations of Api (0.5, 1.25, 2.5, 5, 10, 20, 40 and 80 µM) were prepared using a running buffer. The specialized PlexArray HT software (Plexera Bioscience, Seattle, WA, US) was employed to obtain and analyze the reaction signal.

### Molecular Docking Analysis

The crystal structure of the FGR complex were obtained from the protein data bank (PDB ID: 7UY0), and the structure of Api was obtained from PubChem (CID: 5280443). To analyze potential binding sites between Api and FGR, Schrodinger‐Maestro software (version 11.1) was utilized. The ligand and receptor input files underwent processing with “LigPrep” and “Protein Preparation Wizard”. Receptor grid generation was employed to create a box that encompassed the entire binding site for docking ligand. The docking process followed, with default parameters being utilized.

### Statistical Analysis

The numbers of individual animals or samples used per group or independent experiments are described in each individual figure panel. Each dot corresponds to one mouse or a sample, unless otherwise noted. Unless otherwise stated, data are presented as the mean ± s.e.m. The differences between two groups were compared using an unpaired Student's *t*‐test or Mann‐Whitney U test. One‐way ANOVA with Sidak's multiple comparison was used to evaluate experiments containing more than two groups unless otherwise stated. The correlation coefficients were determined using Spearman's correlation analysis. GraphPad Prism version 9.5 was used for statistical analysis. *p* values less than 0.05 were considered statistically significant and denoted by an asterisk (*).

## Conflict of Interest

The authors declare no conflict of interest.

## Author Contributions

P.G., R.W., R.L., Q.Y., and Y.H. contributed equally to this work. P.G., R.W., R.L., Q.Y., and Y.H. performed experiments and analyzed the data. J.G., W.H., J.L., Y.Z., L.X., J.H., Q.G., and J.H. perform experiments. J.B., W.W., J.G., Z.Z., and Z.C. provided technical support and helped with human samples collection. P.G., Y.J., Z.L., and P.C. designed the study, interpreted the data, drafted and edited the manuscript, and also supervised the study.

## Supporting information



Supporting Information

Supporting Information

## Data Availability

The data that support the findings of this study are available from the corresponding author upon reasonable request.
