# Supplementary material for: Aging‐induced Alternation in the Gut Microbiota Impairs Host Antibacterial Defense
Source: Adv Sci (Weinh). 2025 Jan 10;12(12):2411008. doi: 10.1002/advs.202411008 (PMC11948050; doi:10.1002/advs.202411008)
Supplement: Supplementary file 1 — Supporting Information [file ADVS-12-2411008-s002.docx]

**Supporting Information**

**Aging-induced Alternation in the Gut Microbiota Impairs Host Antibacterial Defense**

Peng Gu^1,2,#^, Rongjuan Wei^2,#^, Ruofan Liu^2,#^, Qin Yang^2,3,#^, Yuxuan He^1,#^, Jianbin Guan^1^, Wenhao He^2^, Jiaxin Li^2^, Yunfei Zhao^2^, Li Xie^2^, Jie He^1^, Qingling Guo^1^, Jiajia Hu^2^, Jingna Bao^4^, Wandang Wang^5^, Jiayin Guo^6^, Zhenhua Zeng^4^, Zhongqing Chen^4^, Yong Jiang^2,7*^, Zhanguo Liu^1,*^, Peng Chen^1,2,*^

^1^Department of Critical Care Medicine, Zhujiang Hospital, Southern Medical University, Guangzhou, 510280, China.

^2^Department of Pathophysiology, Guangdong Provincial Key Laboratory of Proteomics, School of Basic Medical Sciences, Southern Medical University, Guangzhou, 510515, China.

^3^Department of Gastroenterology, The Seventh Affiliated Hospital of Southern Medical University, Foshan, 528244, China.

^4^Department of Critical Care Medicine, Nanfang Hospital, Southern Medical University, Guangzhou, 510510, China.

^5^Department of Clinical Medicine Laboratory, Affiliated Xiaolan Hospital, Southern Medical University, Zhongshan, 528415, China.

^6^NMPA Key Laboratory for Research and Evaluation of Drug Metabolism, Guangdong Provincial Key Laboratory of New Drug Screening, School of Pharmaceutical Sciences, Southern Medical University, Guangzhou, 510515, China.

^7^Department of Respiratory and Critical Care Medicine, The Tenth Affiliated Hospital, Southern Medical University, Dongguan, 523059, China.

**Materials and Methods**

**Cells and Cell Lines**

Bone marrow-derived macrophages (BMDMs) were isolated from mouse tibia and femur and cultured in DMEM containing 1% penicillin-streptomycin, 10% fetal bovine serum (FBS), and 20 ng/mL macrophage colony-stimulating factor (Miltenyi, 130-101-705). Following a week of culture, the cells were prepared for subsequent experiments ^[1]^ .

THP1 cells (ATCC TIB-202) were grown in 1640 medium containing 1% penicillin-streptomycin and 10% FBS. Following that, the cells were induced to differentiate into macrophages (THP1-dMs) by treating them with phorbol 12-myristate 13-acetate (5 nM, MCE, HY-18739) for a duration of 48 h.

The 6% broth-elicited peritoneal macrophages were prepared from AM as previously described ^[2]^ . Cells were cultured in RPMI 1640 medium containing 10% FBS and 1% penicillin-streptomycin for 4 h, and washed with the medium to remove non-adherent cells.

Neutrophils were isolated from mouse bone marrow ^[3]^ . Briefly, bone marrow cells from mouse femurs and tibias were harvested and resuspended in 1-3 mL of ice-cold sterile PBS. To purify the neutrophils, 3 mL of Histopaque 1119 (density, 1.119 g/mL, Sigma-Aldrich, 11191) and 3 mL of Histopaque 1077 (density, 1.077 g/mL, Sigma-Aldrich, 10771) were cautiously added into a 15 mL conical centrifuge tube and overlay with 1mL of the bone marrow cell suspension. After centrifuging at 800 × g for 30 minutes at room temperature, neutrophil-enriched layers were collected and subjected to two washes with PBS at 200 × g for 5 minutes each time. Finally, neutrophils were cultured in 1640 medium consisting of 10% FBS and 1% penicillin-streptomycin.

**Bacterial Strains**

*E. coli* (WT strain ATCC 25922) was grown in LB medium at 37 °C until the optical density reached 1 (OD600 = 1) and diluted to the appropriate concentration in sterile PBS. *P. goldsteinii* (JCM 13446) were grown on anaerobic blood agar or fluid thioglycolate medium at 37 °C in an anaerobic chamber filled with a mixture of anaerobic gases. Prior to oral gavage, *P. goldsteinii* cells were harvested through centrifugation and subsequently resuspended in a sterile saline solution. Pasteurized *P. goldsteinii* were prepared by heating the bacteria at 70 °C for 60 minutes. The mutant strain of *P. goldsteinii* was generated by homologous recombination as previously described, with slight modification ^[4]^ . Briefly, a recombinant plasmid pUC57-*ermE* containing 1500 bp flanking regions of *ampB* and a selective marker of erythromycin was transformed into competent *P. goldsteinii* cells by electroporation. The electroporated *P. goldsteinii* were added into recovery media and cultured in blood agar plates supplemented with erythromycin (25 μg/mL). Resistant colonies were subsequently selected for identification by PCR using primers specific to the upstream and downstream flanking regions. To assess the growth curve, 200 μL of either the WT or mutant *P. goldsteinii* culture was used to measure optical density at 600 nm (OD600).

**CLP or *E. coli*-induced Bacterial Infection Model of Mouse**

The cecal ligation and puncture (CLP) procedure was executed in accordance with the procedure mentioned in a previous study with slight modifications ^[5]^ . In short, the mice were profoundly anesthetized, and the distal half of the caecum was ligated. A single puncture was created by inserting an 18G needle through-and-through, followed by expulsion of a small amount of fecal matter through the punctured areas. Subsequently, the peritoneum was closed and the mice were revived through subcutaneous administration of 1 mL of sterile saline. To maintain consistency in the source of infection, mice that had undergone FMT or microbiota colonization were intraperitoneally injected with *E. coli* (1 × 10^8^ CFUs per mouse) to construct a bacterial infection model. The surface temperature of the mouse sternum was assessed using an infrared thermometer, as suggested in a previous report ^[6]^ . Mice that could not return to a sternal recumbent position after being placed on their sides were deemed moribund and subsequently euthanized. All survival experiments with mice were monitored for 72 h. The mortality outcomes observed during these experiments stemmed from both the natural progression of diseases and the humane endpoints established for euthanasia. For detecting bacterial load and multiple organ injury, mice were euthanized at the humane endpoint or at the study endpoint (12 h after CLP operation or *E. coli* injection) in each separate cohort of mice.

**Fecal Microbiome Transplantation**

FMT experiment in AM, at least 1 g feces collected from either young or aged donors (both mice and human) were thawed and then homogenized in PBS at a concentration of 0.125 g/mL using a vortex. Following homogenization, the mixture was centrifuged for 5 minutes at 600 × g at a temperature of 4 °C. After centrifugation, the supernatant was carefully transferred to a fresh tube and stored at -80 °C until it was ready for use. For the recipient mice, we administered a pre-treatment consisting of oral gavage of antibiotics cocktail (ampicillin - 200 mg/kg, vancomycin - 100 mg/kg, neomycin - 200 mg/kg, or metronidazole - 200 mg/kg) for a duration of 5 days to deplete gut microbiota. Once the pre-treatment phase was complete, the actual FMT procedure was carried out with the administration of 150 µL fecal suspension through oral gavage once a day for 5 consecutive days. Each mouse received a daily dosage of the fecal suspension to ensure a consistent delivery of the transplanted microbiota.

**Microbial Colonization**

For gnotobiotic colonization of human microbiota, the stool was thawed, washed with sterile PBS, and diluted in PBS with 15% glycerol. 150 µL of the suspension was orally gavaged once a day to GF mice housed in isocages for 7 days. Subsequently, further experiments were initiated.

For bacterial colonization, WT and *ampB*-knockout *P. goldsteinii* were cultivated anaerobically in fluid thioglycolate medium. Subsequently, the bacterial cultures were purified and concentrated in anaerobic PBS to achieve a final concentration of 1 × 10^9^ CFUs per mL, ensuring strict anaerobic conditions. The SPF mice and GF mice were administered with 2 × 10^8^ CFUs of viable *P. goldsteinii*, pasteurized *P. goldsteinii,* or *ampB*-knockout *P. goldsteinii* in 200 μL of sterile anaerobic PBS through gavage on a daily basis for 5 consecutive days.

**Quantitative Analysis of Api**

A liquid chromatography-tandem mass spectrometry system (LC-MS/MS) was utilized to detect Api levels. The cecal content samples were added with a 9-fold volume/weight of water and subjected to ultrasonication for 10 minutes at 4 °C. To precipitate the proteins, methanol (1:4, v/v) was added to 200 μL of homogeneous cecal content or 100 μL of plasma. Following this, the solution was centrifuged at 15,000 × rpm for 10 minutes at 4 °C. The supernatant was dried by a nitrogen blowing instrument and subsequently redissolved in 300 μL methanol. Finally, the solution was centrifuged for 10 minutes at 15,000 × rpm and 4 °C to collect the supernatant, which was later analyzed using LC-MS/MS. The specific parameters for analysis are comparable to those outlined in nontarget metabolomics.

**Bacterial Load Analysis**

In order to analyze the bacterial load, various samples including blood, peritoneal lavage fluid (PLF), liver, and spleen were collected. The solid tissue specimens were accurately weighed, then homogenized in PBS solution. Following this, the homogenate was separated from the solid particles by subjecting it to centrifugation at room temperature for 1 minute at a speed of 1000 × rpm. The resulting supernatants were then subjected to serial dilution. By taking 100 μL of these diluted samples, each was carefully placed on either aerobic or anaerobic Columbia blood agar plates, as well as LB plates. After an incubation period of approximately 14-16 h at 37 °C, the formed CFUs were counted.

**Blood Routine Parameters**

One hour after *E. coli* injection, at least 200 μL abdominal aorta blood was collected into a heparin-coated tube. Levels of white blood cells (WBC), middle cells (MID), lymphocytes (LYM), and granulocytes (GRAN) were examined using an automatic blood analyzer (Prokan, PE-6800).

**Plasma Biochemistry**

The levels of ALT and AST in mouse plasma were measured by employing commercial ALT kits (Nanjing Jiancheng, C009-3-1) and AST kits (Nanjing Jiancheng, C010-3-1) as per the instructions provided by the manufacturer. The plasma levels of BUN and CREA were detected using an automatic analyzer (Mindray, BS-330E) according to manufacturer's instructions.

**Histological Procedures**

Lung, liver, and kidney tissues of mice were collected and fixed in 4% paraformaldehyde following euthanasia. The tissues were then embedded in paraffin. Tissue sections with a thickness of 5 μm were obtained and subjected to H&E staining. Five fields were randomly chosen from each sample for observation. Lung injury severity was measured with a score from 0 to 3 for each parameter including alveolar congestion, hemorrhage, infiltration by inflammatory cells, and thickening of the alveolar wall ^[7]^ . Liver injury severity was assessed using a scoring system with the highest score of 12 for various parameters, including necrosis, inflammation, ballooning degeneration, and disruption of hepatic cord structure ^[8]^ . The assessment of kidney injury severity was determined based on damages to renal tubules and glomeruli, scored from 0 to 5 ^[9]^ .

**DNA/RNA Isolation and Quantitative PCR**

Total DNA obtained from fecal samples was extracted using the SPINeasy DNA Kit for Feces (MP Biomedicals, 116531050), following the instructions provided by the manufacturer. Macrophages were used to isolate the total RNA by employing the FastPure Cell/Tissue Total RNA isolation kit (Vazyme, RC112-01). The cDNA synthesis was accomplished using the ReverTra Ace® qPCR RT Master Mix (Toyobo, FSQ-101) with the total RNA as the template. The manufacturer's protocol for the SYBR Green RT-PCR Kit (Toyobo, QPK-201) was followed to determine the relative levels of *P. goldsteinii* or Fgr, utilizing the ABI 7500 system (Applied Biosystems). The endogenous control for this study was the transcription of 16S or 18S. Refer to Supplementary Table S3 for the primer sequence information used in this study.

**CCK-8 Assay**

To evaluate the viability of the cells, the Cell Counting Kit-8 (CCK-8, Dojindo, CK04) was utilized in accordance with the guidelines provided by the manufacturer. Initially, the cells were seeded in 96-well plates at a density of 10^5^ cells per well, following which they were subjected to various concentrations of Api (0, 0.1, 1, 10, 20, 30, 40, and 50 μM) for a duration of 3 h. Subsequently, the CCK8 reagent was introduced and allowed to incubate for 1 h at a temperature of 37 °C with 5% CO_2_. The absorbance of the cells at 450 nm was measured utilizing a microplate reader (SpectraMax M5).

**Flow Cytometry**

pHrodo red *E. coli* BioParticles (Thermo Fisher, P35361, 200 μL) were injected into the peritoneal cavity of DMSO- or Api-treated AM. Mice were sacrificed 1 h after injection. 10 mL of pre-warmed PBS was injected into the abdominal cavity of AM and aspirated out after gently pressing to collect the peritoneal lavage fluid. Peritoneal cells were collected by centrifugating at 1000 × rpm for 10 minutes and stained with fluorochrome-conjugated monoclonal antibodies against mouse F4/80 (Invitrogen, 48-4801-80) and CD11b (BD Pharmingen, 557396) as described previously ^[10]^ . After wash with PBS, fluorescent cells were detected using a BD LSRFortessa^TM^ X-20 Cell Analyzer and analyzed using FlowJo software (Tree Star Inc.).

**Phagocytosis Assay**

The CFUs counting analysis involved a pretreatment of macrophages with a concentration of 20 μM of Api for a duration of 3 h. Subsequently, the macrophages were incubated with *E. coli* at 37 °C in an environment containing 5% CO_2_ for a period of 45 minutes. The infection was carried out at a multiplicity of infection (MOI) of 50. Following the 45-minute incubation, the macrophages underwent two washes with PBS supplemented with gentamycin at a concentration of 0.5 µg/mL to eliminate any extracellular bacteria. In a separate experiment, a suspension containing 10^6^ neutrophils was transferred to 24-well plates containing 1640 medium. The neutrophils were treated with either DMSO or a concentration of 20 µM of Api for a duration of 3 h. Subsequently, the neutrophils were incubated with *E. coli* at a ratio of 1:100 (MOI = 1:100) in a controlled environment at a temperature of 37 °C for a period of 45 minutes. To separate the neutrophils from the bacteria in the suspension, centrifugation was performed at a force of 400 × g for 3 minutes at a temperature of 4 °C. The resulting pellet containing the neutrophils was suspended in 1640 medium supplemented with gentamicin at a concentration of 50 μg/mL for a duration of 30 minutes to eliminate any adherent extracellular bacteria. Finally, the cells were lysed with a solution containing 0.1% TritonX-100 to release the intracellular bacteria. The released bacteria were then plated on LB agar medium and incubated overnight to facilitate the determination of CFUs counts.

**Immunoprecipitation Assays**

Fgr-Flag (wild-type, M341A and D404A) plasmids and Syk-HA plasmid were synthesised by GENEYUAN Bio-Tec. After transfection for 48 h, the THP1-dMs were pretreated with Api (20μM) for 3 h and then stimulated with *E. coli*. Immunoprecipitation was executed according to the instructions of the BeyoMag™ Anti-Flag Magnetic Beads (Beyotime, P2115). Briefly, cells were washed once with PBS and then lysed in immunoprecipitation lysate containing a freshly added protease inhibitor for 10 minutes on ice. The lysates from the cells were subjected to centrifugation to eliminate any cellular debris. The resulting supernatant was subsequently incubated with 20 μL of anti-FLAG beads overnight at a temperature of 4 °C while maintaining a constant rotation. Washing the beads thoroughly three times using TBS. Finally, the beads were boiled with 1× SDS sample buffer for 5 minutes and subjected to western blotting.

**Western Blotting**

To conduct the activated Rac1 and Cdc42 assay, we followed the protocol provided by the manufacturer (Cytoskeleton, BK034 & BK035). Initially, cells were treated as indicated and exposed to *E. coli* for a duration of 10 minutes. Subsequently, PBS washes were performed, and the cells were collected in ice-cold cell lysis buffer containing 1 × protease inhibitor cocktail. This cell suspension was then centrifuged at 10000 × g at 4 °C for 15 minutes. Protein concentrations were determined using the Pierce™ BCA protein assay kit (Thermo Fisher, 23225). For each sample, 10 μL of PAK- PBD beads were combined with a total of 500 μg proteins, followed by incubation on a rotator at 4 °C for 1 h. After a centrifugation step at 5000 × g at 4 °C for 1 minute and a single wash with 500 μL of wash buffer, the supernatants were eliminated by centrifugation at 5000 × g at 4 °C for 3 minutes. Eventually, the beads were eluted with 20 μL of 2 × laemmli sample buffer and subsequently boiled for 2 minutes. The resulting sample was subjected to 12% SDS–PAGE for further analysis.

Protein extraction from cells was conducted using RIPA lysis buffer (Beyotime, P0013B) supplemented with a protease inhibitor cocktail. The extracted proteins were then subjected to SDS-PAGE (7.5%-15%) for separation. Subsequently, the protein content was transferred onto polyvinylidene difluoride (PVDF) membranes (Merck Millipore, Billerica, MA, USA) and allowed to transfer for approximately 1–2 h. The transferred proteins on the membranes were then blocked with Quick Block Buffer (Beyotime, P0252) at room temperature for 25 minutes. Following the blocking step, the membranes were incubated overnight at 4 °C with the primary antibodies specified. TBST was used to wash the membranes, after which they were incubated with a secondary antibody for 1 h at room temperature. Detection of protein band signals was achieved using ECL reagent and visualized using a ChemiDoc MP imaging system (BIORAD). Analysis of the blotted bands was performed using ImageJ software.

Detailed antibody information was as follows: anti-Fgr antibody (Boster, A01674-1); anti-Flag antibody (Solarbio, K200001M); anti-HA antibody (Solarbio, K200003M); anti-Vav1 antibody (Cell Signaling Technology, 2502), anti-Phospho-Vav1 antibody (Affinity, AF3182), anti-Rac1 antibody (Cytoskeleton, ARC03); anti-Cdc42 antibody (Cytoskeleton, ACD03); anti-Arp2 antibody (Cell Signaling Technology, 3128); anti-Arp3 antibody (Cell Signaling Technology, 4738); anti-Gapdh antibody (Proteintech, 60004) ; anti-rabbit IgG, HRP-linked antibody (Cell Signaling Technology, 7074); anti-mouse IgG, HRP-linked antibody (Cell Signaling Technology, 7076); anti-mouse IgG for IP (Vazyme, RA1009).

**Reference**

[1] T. Lei, J. Zhang, Q. Zhang, X. Ma, Y. Xu, Y. Zhao, L. Zhang, Z. Lu, Y. Zhao, *Cell Mol Immunol* **2022**, *19*, 1333.

[2] A. De Jesus, C. M. Pusec, T. Nguyen, F. Keyhani-Nejad, P. Gao, S. E. Weinberg, H. Ardehali, *STAR Protoc* **2022**, *3*, 101668.

[3] M. Swamydas, Y. Luo, M. E. Dorf, M. S. Lionakis, *Curr Protoc Immunol* **2015**, *110*, 3.

[4] S. K. Sharan, L. C. Thomason, S. G. Kuznetsov, D. L. Court, *Nat Protoc* **2009**, *4*, 206.

[5] D. Rittirsch, M. S. Huber-Lang, M. A. Flierl, P. A. Ward, *Nat Protoc* **2009**, *4*, 31.

[6] O. Laitano, D. Van Steenbergen, A. J. Mattingly, C. K. Garcia, G. P. Robinson, K. O. Murray, T. L. Clanton, E. A. Nunamaker, *Shock* **2018**, *50*, 226.

[7] P. Gu, R. Liu, Q. Yang, L. Xie, R. Wei, J. Li, F. Mei, T. Chen, Z. Zeng, Y. He, H. Zhou, H. Peng, K. S. Nandakumar, H. Chu, Y. Jiang, W. Gong, Y. Chen, B. Schnabl, P. Chen, *Cell Mol Immunol* **2023**, *20*, 1156.

[8] Q. Zhang, J. Wei, Z. Liu, X. Huang, M. Sun, W. Lai, Z. Chen, J. Wu, Y. Chen, X. Guo, Q. Huang, *Redox Biol* **2022**, *54*, 102367.

[9] J. Dawulieti, M. Sun, Y. Zhao, D. Shao, H. Yan, Y. H. Lao, H. Hu, L. Cui, X. Lv, F. Liu, C. W. Chi, Y. Zhang, M. Li, M. Zhang, H. Tian, X. Chen, K. W. Leong, L. Chen, *Sci Adv* **2020**, *6*, eaay7148.

[10] M. Zhou, M. Aziz, H. T. Yen, G. Ma, A. Murao, P. Wang, *Cell Mol Immunol* **2023**, *20*, 80.


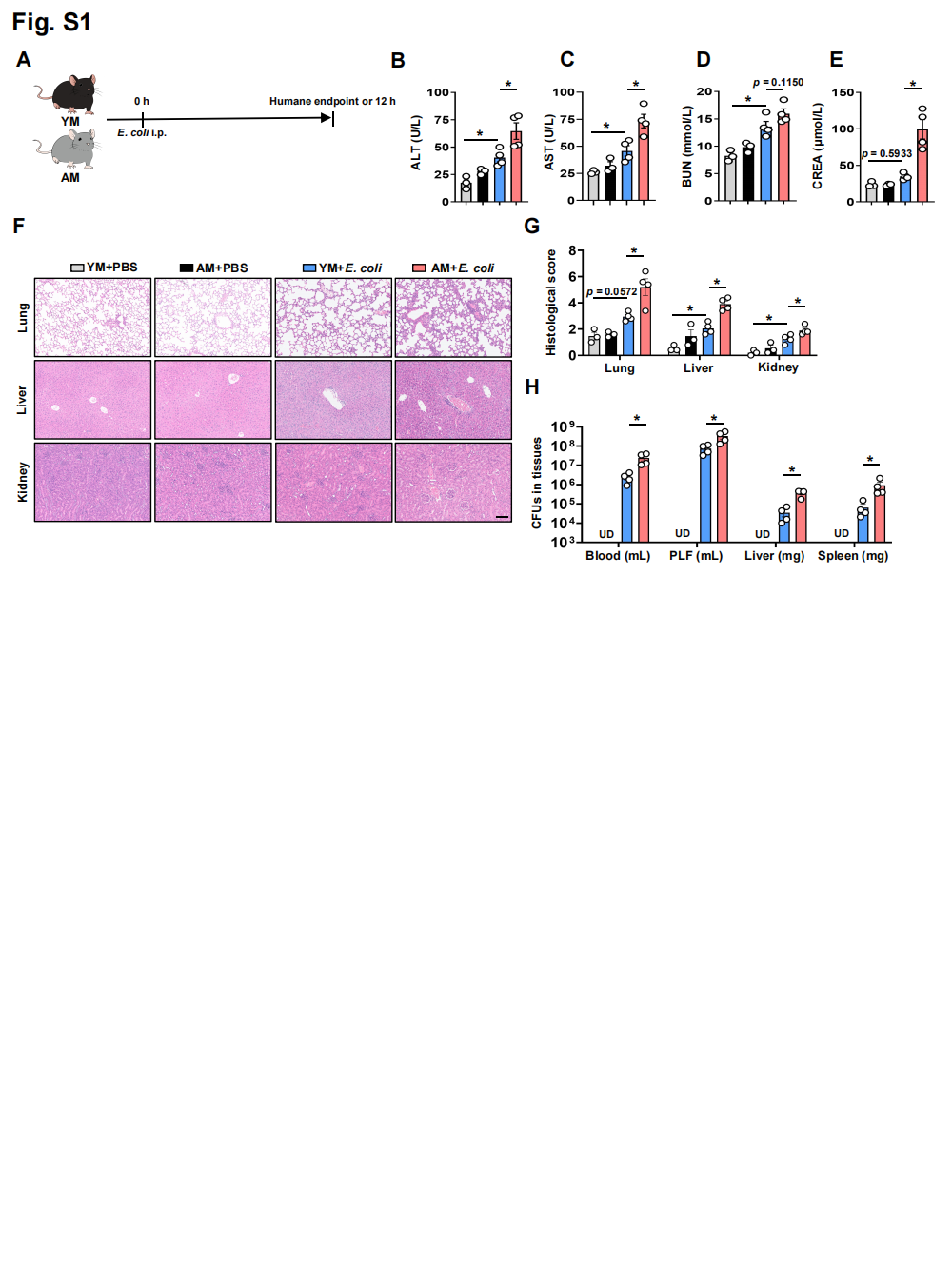


**Figure S1. Aged mice showed worse bacterial infection outcomes.** A) Experimental design for bacterial infection by intraperitoneal injection of *E. coli* in YM and AM. B-E)**.** Plasma levels of ALT, AST, BUN, and CREA. n = 3-4. F) Representative H&E staining of lungs, liver, and kidneys. Scale bar: 100 μm. G) Pathological scores of lungs, liver, and kidneys. n = 3-4. H) *E. coli* burden in blood, PLF, liver, and spleen under aerobic condition. n = 3-4. Data are presented as mean ± s.e.m. Statistical analysis was performed using One-way ANOVA with Sidak’s multiple comparison. *, *p* < 0.05. UD: undetected.


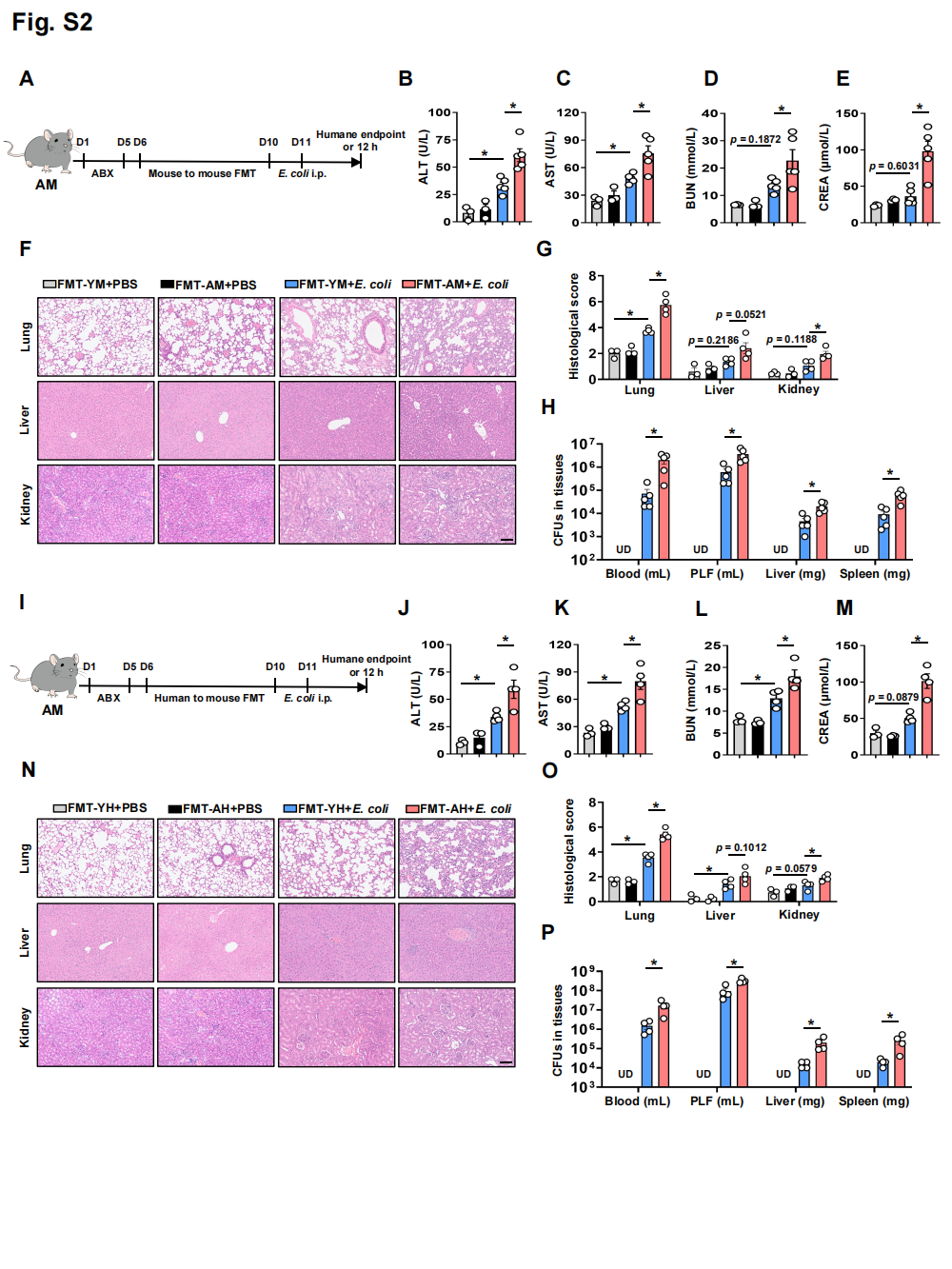


**Figure S2. Increased susceptibility of bacterial infection in aged groups is associated with gut microbiota.** A) The fecal supernatant from young or aged donor mice was transplanted to antibiotic-treated recipient AM, followed by injection of *E. coli*. B-E) The plasma ALT, AST, BUN, and CREA levels. n = 3-5. F, G) Pathological changes and scores in lungs, liver, and kidneys were indicated by H&E staining. n = 3-4. Scale bar: 100 μm. H) *E. coli* colonies in blood, PLF, liver, and spleen. n = 3-5. I) Experimental design for the ABX treatment and transplantation of human fecal microbiota to AM model. J-M) Plasma ALT, AST, BUN, and CREA concentration. n = 3-4. N, O) H&E-stained histological images and scores in lungs, liver, and kidneys. n = 3-4. Scale bar: 100 μm. P) Quantification of *E. coli* colonies in the blood, PLF, liver, and spleen. n = 3-4. Data are presented as mean ± s.e.m. Statistical analysis was performed using One-way ANOVA with Sidak’s multiple comparison. *, *p* < 0.05. UD: undetected.


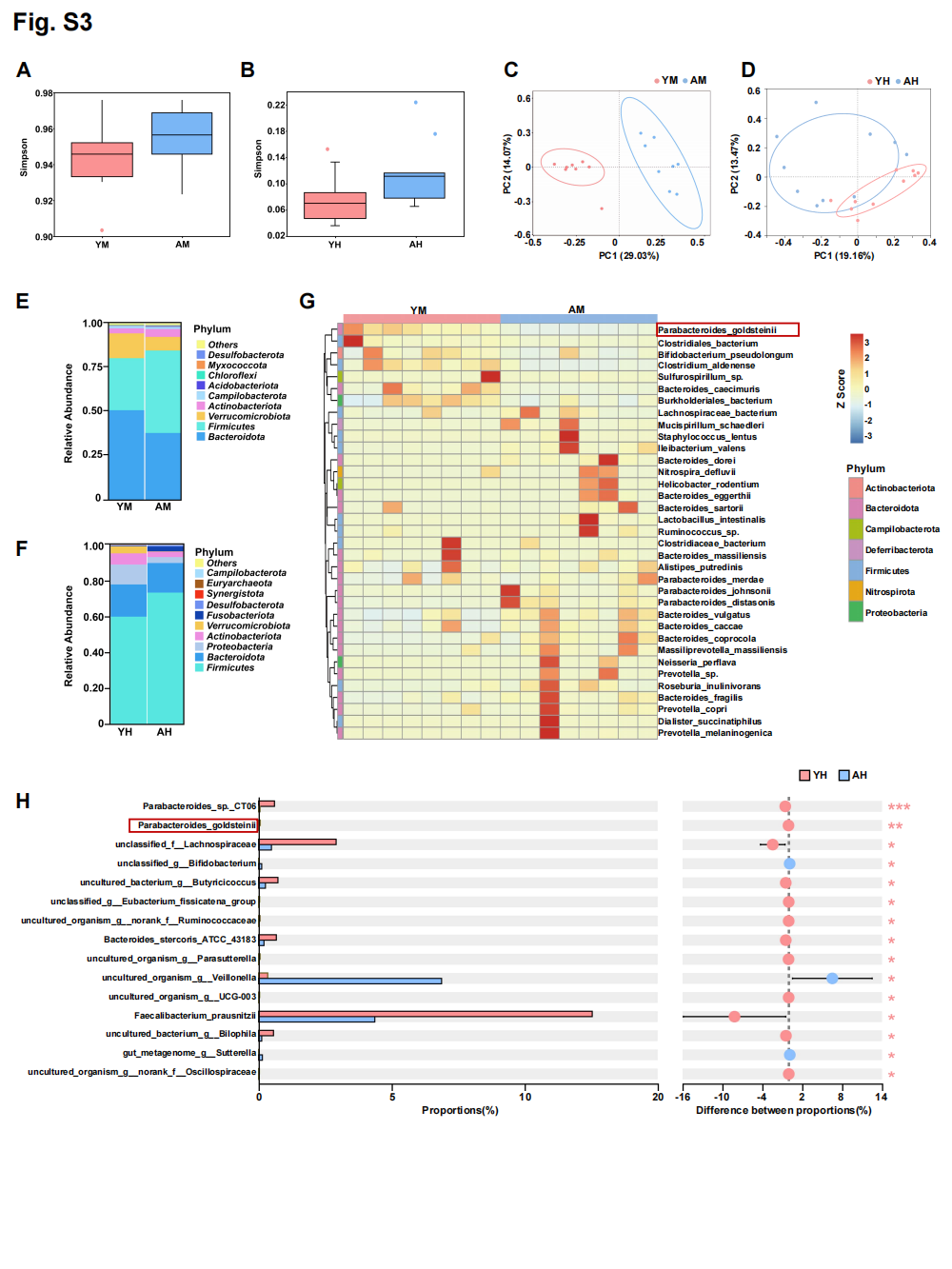


**Figure S3. Aging-related alteration in commensal microbiota composition.** A, B) α-diversity of the gut microbiota between the young and aged mice (A, n = 8) and humans (B, n = 10) as indicated by the Simpson index. C, D) Principal component ordination (PcoA) analysis using the Bray-Curtis distance. For mice, n = 8; for human, n = 10. E, F) Phylum-level histograms show the different composition of gut microbiota in young and aged mice (E, n = 8) and humans (F, n = 10). G) Heatmap representation of the difference in gut microbiome between YM and AM at the species level. Red and blue represent high and low abundance, respectively. n = 8. H) Comparison of relative abundance of different gut microbiota between young and aged humans. n = 10. Statistical analysis was performed using two-tailed Student’s t-tests. *, *p* < 0.05, **, *p* < 0.01，***, *p* < 0.001


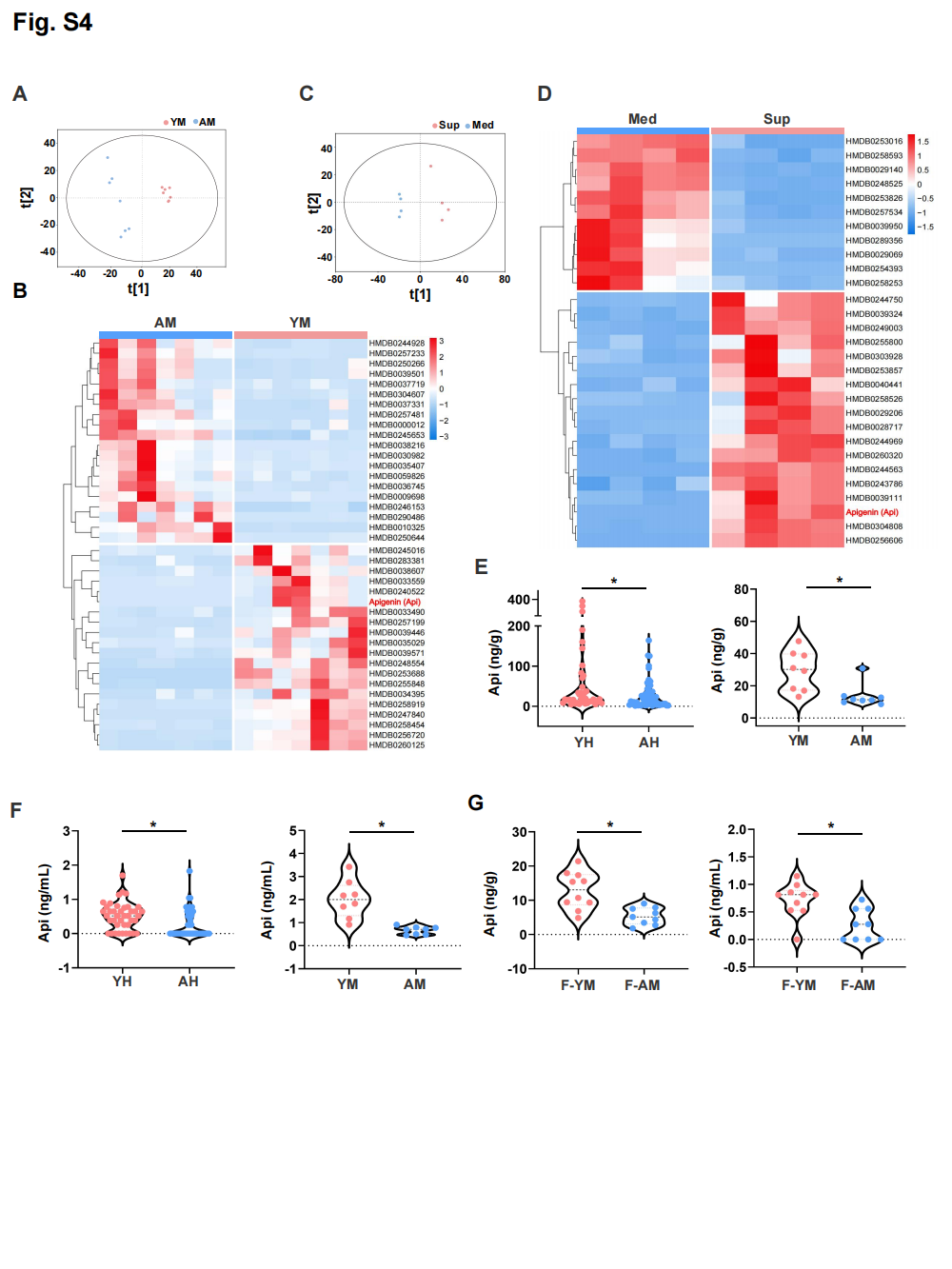


**Figure S4. Identification of gut metabolites alterations.** A) PLSDA plot for gut metabolomics analysis of YM and AM. n = 7. B) Heatmap analysis of different metabolites in fecal of YM and AM. n = 7. C) PLSDA analysis revealed clear separations among blank medium and *P. goldsteinii* culture supernatant. n = 4. D) Heatmap analysis of blank medium and *P. goldsteinii* culture supernatant. n = 4. E) Api concentration in fecal samples of young and aged human and mice. For human samples, n = 40-56; for mice, n = 8. F) Plasma Api concentration between young and aged human and mouse individuals. For humans, n = 40-56; for mice, n = 8. G) YM and AM were fed with flavonoid-free diet for 2 weeks. Api concentration in feces and plasma samples were determined by LC-MS/MS. n = 9-10. Data are presented as median ± quartiles; Statistical analysis was performed using two-tailed Student’s t-tests. *, *p* < 0.05.


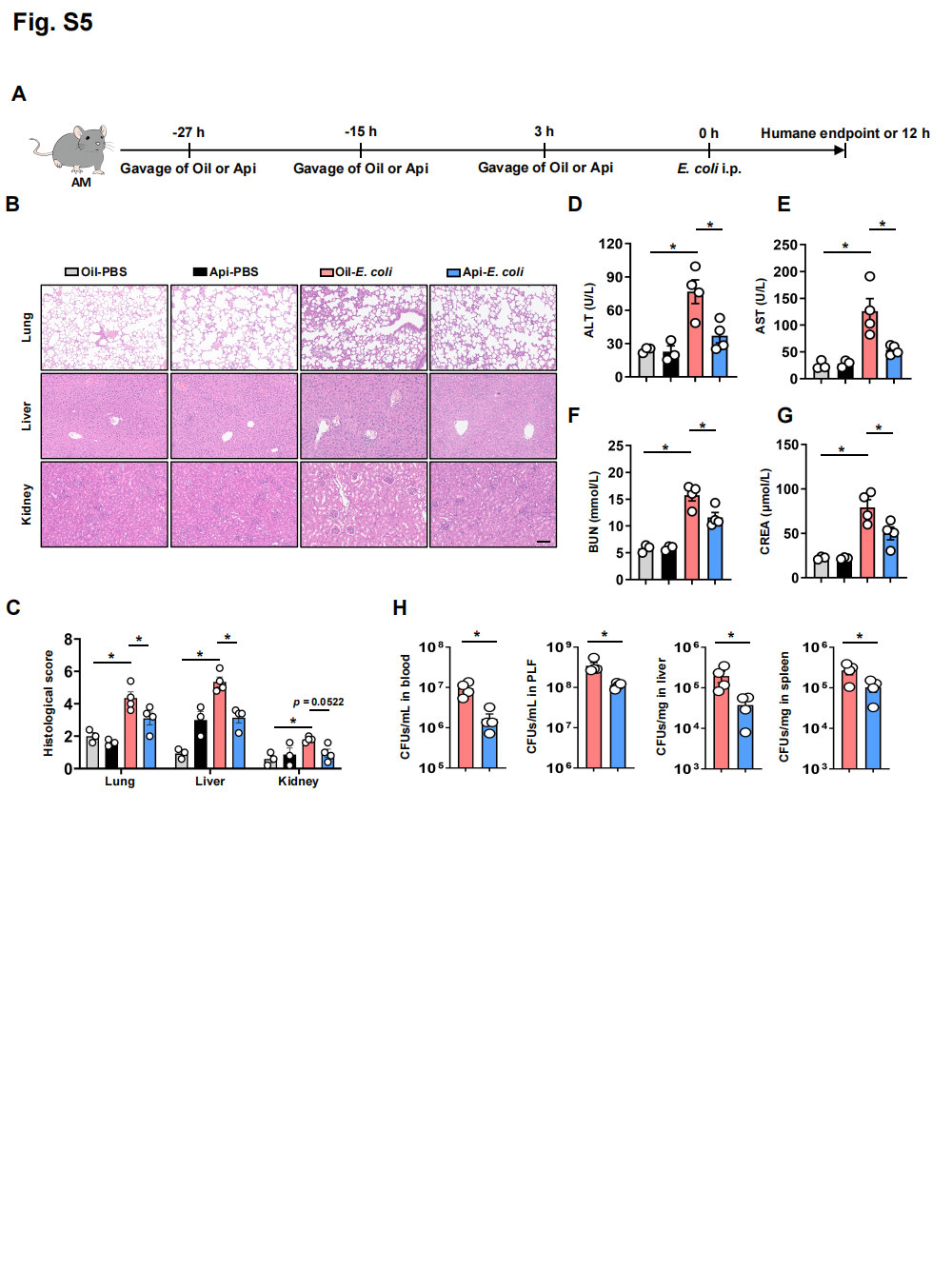


**Figure S5. The protective effect of apigenin on bacterial infection in AM.** A) AM were gavaged with oil or Api for 3 times every 12 h before intraperitoneal injection of *E. coli*, the tissues were collected for analysis. B) Representative H&E staining images for lungs, liver, and kidneys from oil and Api-treated mice. Scale bar: 100 μm. C) Pathological scores of lungs, liver, and kidneys. n = 3-4. D-G) Plasma levels of ALT, AST, BUN, and CREA. n = 3-4. H) Bacterial load in blood, PLF, liver, and spleen. n = 4. Data are presented as mean ± s.e.m. Statistical analysis was performed using two-tailed Student’s t-tests or One-way ANOVA with Sidak’s multiple comparison. *, *p* < 0.05.


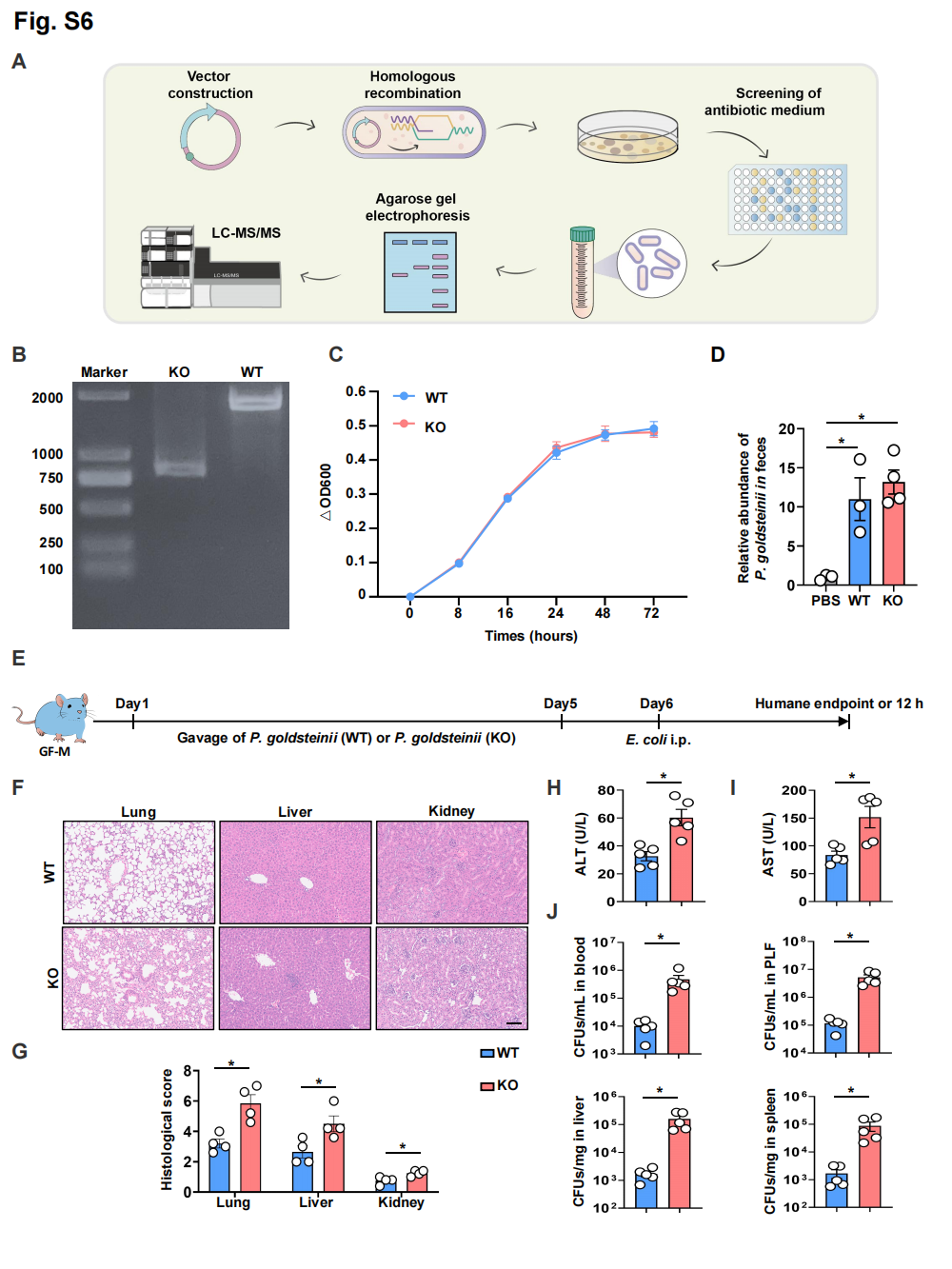


**Figure S6. Commensal *P. goldsteinii* produces the apigenin in the presence of *ampB*.** A) Schematic diagram illustrating the workflow for *ampB* gene deletion in *P. goldsteinii*. B) Deletion of the *ampB* gene in *P. goldsteinii* was validated by PCR. C) Growth curves of WT and *ampB*-KO *P. goldsteinii*. n = 5. D) The relative abundance of wild-type and mutant *P. goldsteinii* in feces were determined by qPCR. n = 3-4. E) Schematic diagram shows the experimental design and timeline in GF mice. F) Representative H&E-stained histologic images of lung, liver, and kidney tissues from GF mice. Scale bar: 100 μm. G) Pathological scores of H&E-stained histologic images. n = 4. H, I) Plasma ALT and AST levels in GF mice treated WT- or *ampB*-KO *P. goldsteinii*. n = 5. J) Statistical comparison of bacteria counts in blood, PLF, liver and spleen. n = 5. Data are presented as mean ± s.e.m. Statistical analysis was performed using One-way ANOVA with Sidak’s multiple comparison or two-tailed Student’s t-tests. *, *p* < 0.05.


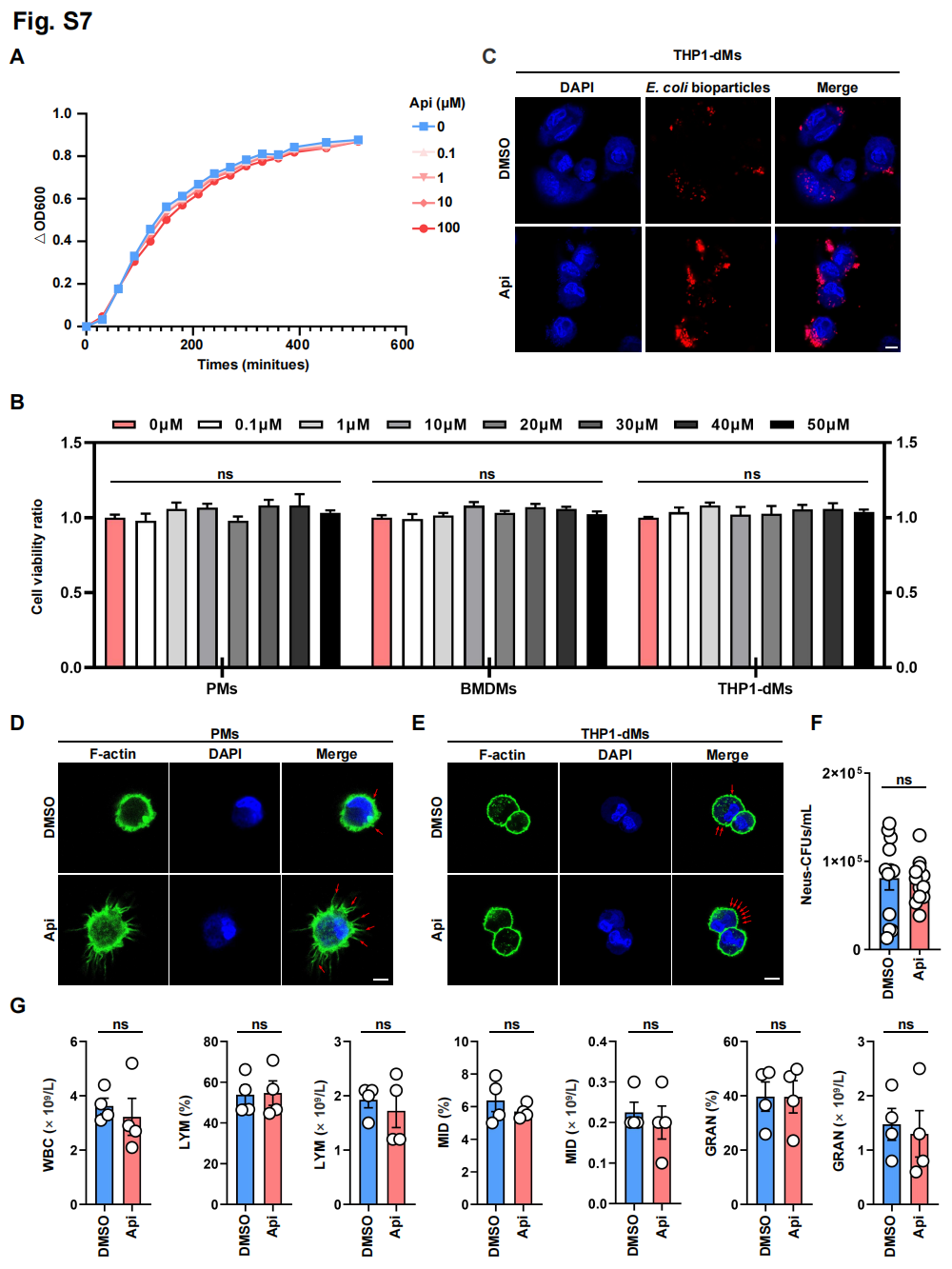


**Figure S7. Dose safety evaluation of Api and its effect on phagocytosis of macrophages and neutrophils.** A) Co-treatment with different concentrations of Api did not markedly influence the proliferation status of *E. coli*. n = 6. B) PMs, BMDMs or THP1-dMs viability was examined by Cell Counting Kit-8 (CCK-8). n = 5-6. C) Representative fluorescence images of DMSO- or Api-treated THP1-dMs with pHrodo red *E. coli* bioparticles and blue (DAPI) nuclear staining over a course of 30 minutes. Scale bar: 10 μm. D, E) PMs (D) and THP1-dMs (E) were pretreated with DMSO or Api, and stimulated with *E. coli* for 45 minutes and stained with phalloidin (green) and Dapi (blue). Cells were examined by laser-scanning confocal microscopy, and representative cells were shown. Scale bar: 10 μm. F) Neutrophils were pretreated with or without Api for 3 h and then infected with *E. coli* for 45 minutes. Quantification results of phagocytosis were presented. n = 12. G) Blood parameters of the Api- or DMSO-treated AM 1 hour after *E. coli* injection. n = 4. Data are presented as mean ± s.e.m. Statistical analysis was performed using One-way ANOVA with Sidak’s multiple comparison or two-tailed Student’s t-tests. ns. not significant. WBC: white blood cell; LYM: lymphocyte; MID: intermediate cell; GRAN: granulocyte.


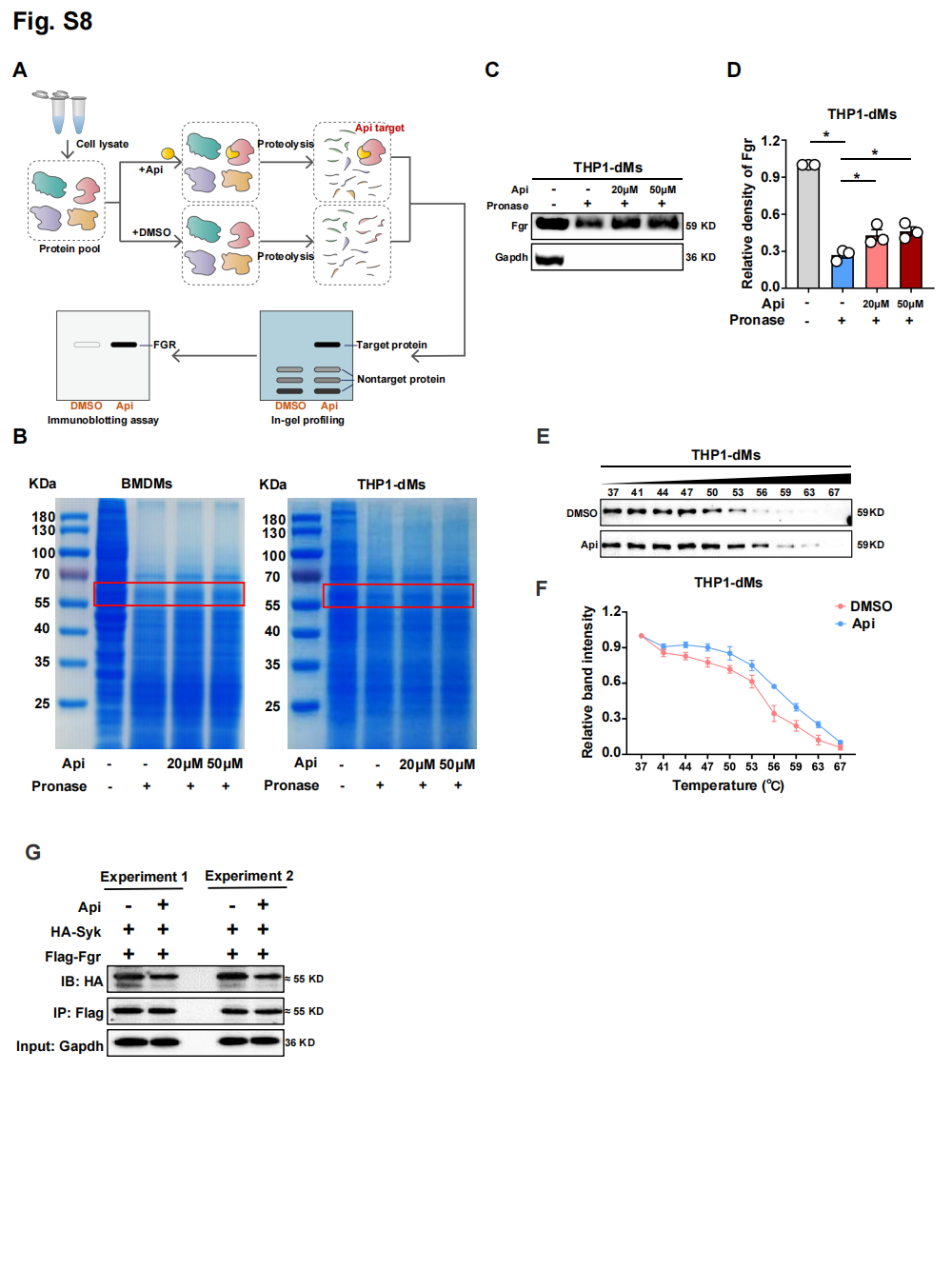


**Figure S8. Identification target protein of Api.** A) Overall schematic diagram showing target identification of Api in macrophage lysates. B) A marked increase in 55-70 kD band upon Api incubation in pronase digested BMDMs or THP1-dMs lysates as indicated by Coomassie Blue staining. C, D) THP1-dMs lysates were treated with DMSO or different concentrations of Api, and then incubated with pronase. The expression of Fgr was detected by immunoblotting. n = 3 independent experiments. E, F) CETSA assay was used to evaluate the binding between Api and Fgr in thermodynamic levels. The representative image of Fgr immunoblotting and quantitative results were presented. n = 3 independent experiments. G) Co-immunoprecipitation analysis of Fgr with Syk. Encoding Flag-tagged Fgr and HA-tagged Syk plasmids were co-transfected into THP1-dMs, and then treated with DMSO or Api for 3 h. Data are presented as mean ± s.e.m. Statistical analysis was performed using One-way ANOVA with Sidak’s multiple comparison. *, *p* < 0.05.


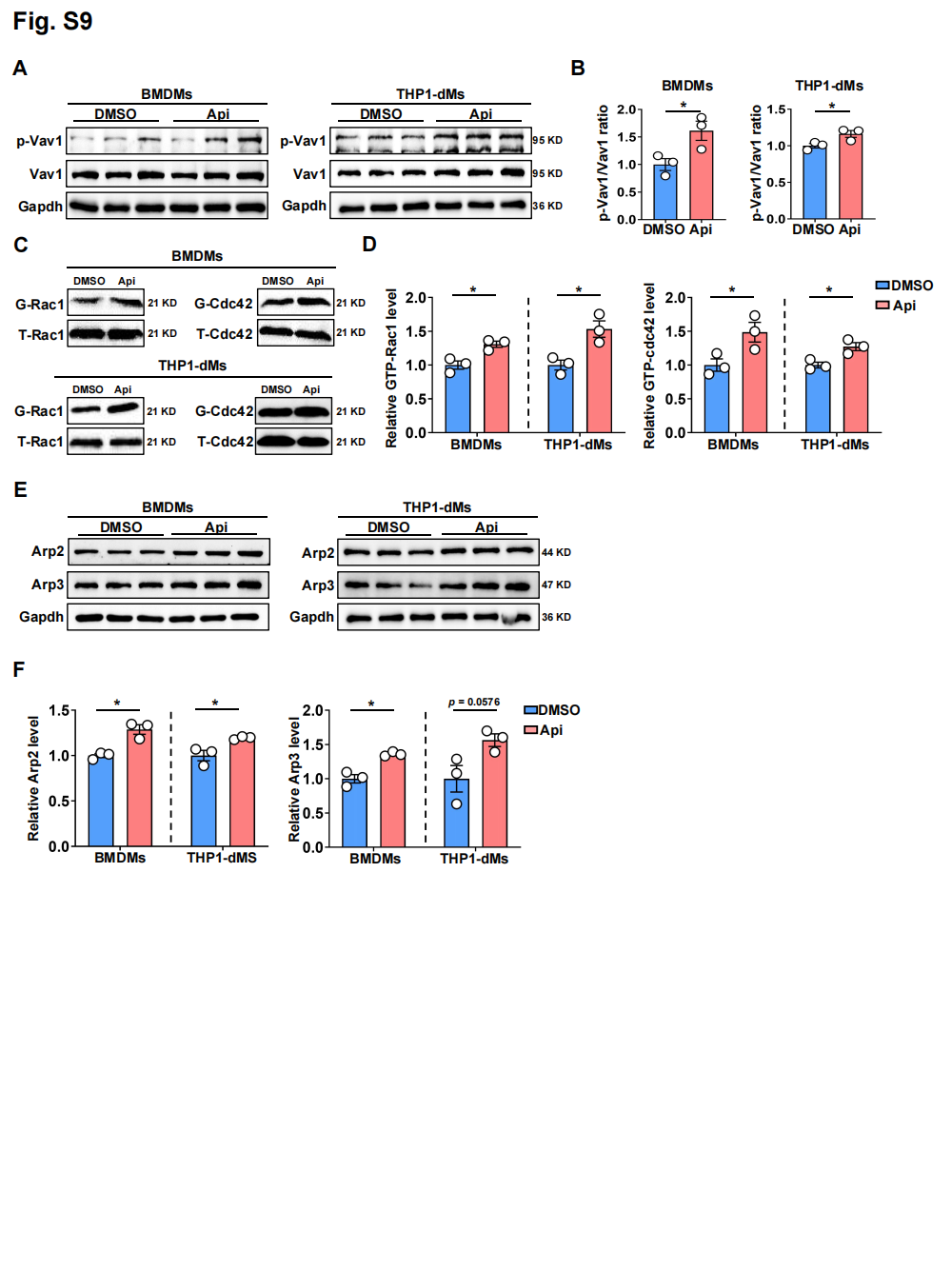


**Figure S9. Api activated Vav1-Rac1/Cdc42-Arp2/3 axis.** A, B) Western blotting analysis and quantitative results showing the phosphorylation of Vav1 after the treatment of Api in BMDMs or THP1-dMs. n = 3 independent experiments. C, D) BMDMs or THP1-dMs were pretreated with DMSO or Api for 3 h and then stimulated with *E. coli*. The GTP-Rac1 or GTP -Cdc42 was enriched via Rac1/Cdc42 activation magnetic beads and measured by western blotting. n = 3 independent experiments. E, F) BMDMs or THP1-dMs were pre-treated with DMSO or Api and stimulated with *E. coli*, and cell lysates were analysed by immunoblotting with anti-Arp2 and anti-Arp3. n = 3 independent experiments. Data are presented as mean ± s.e.m. Statistical analysis was performed using two-tailed Student’s t-tests. *, *p* < 0.05.


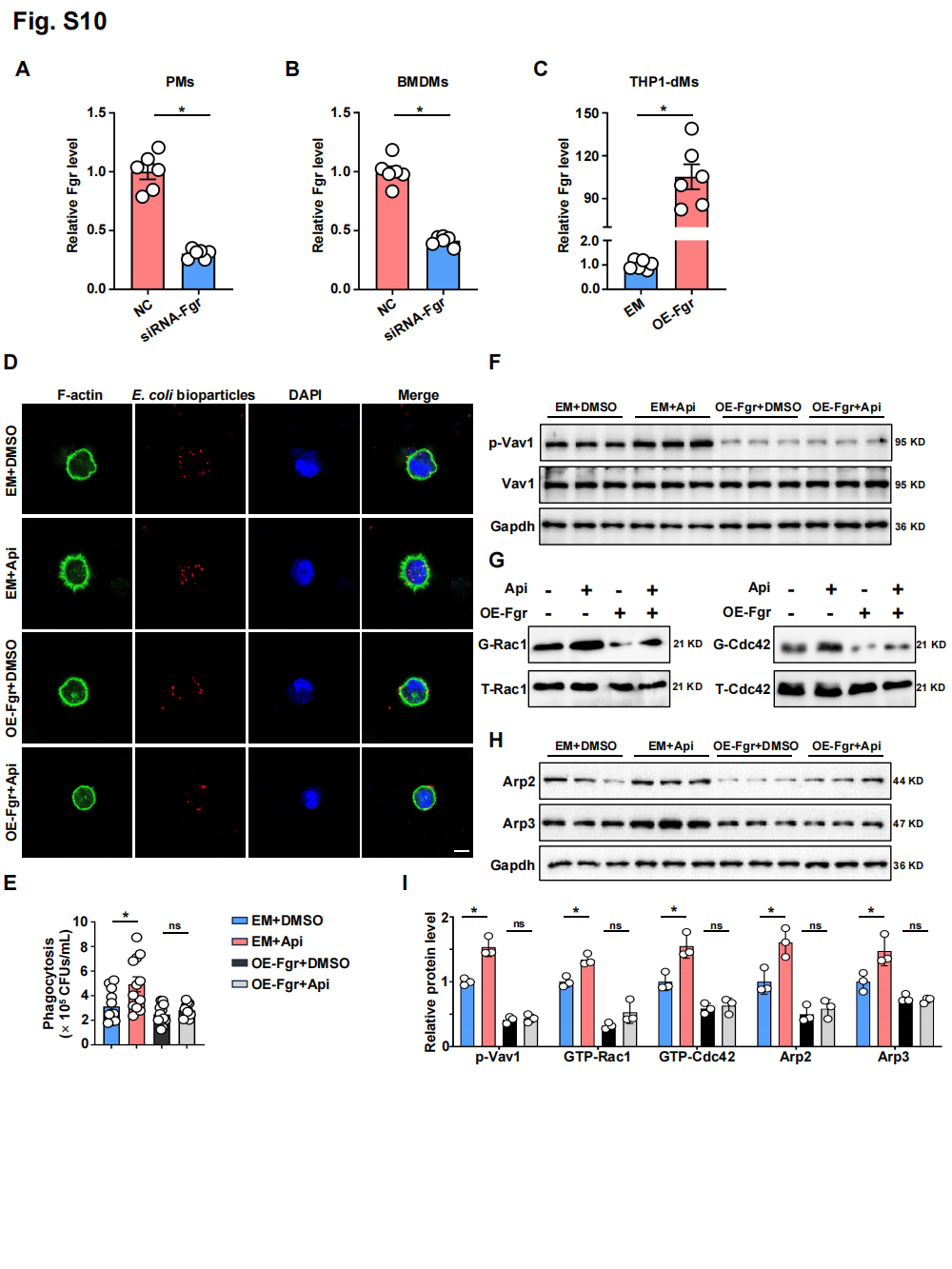


**Figure S10. Api inhibited the binding of Fgr to Syk.** A, B) Relative gene expression of Fgr in PMs (A) and BMDMs (B) transfected with si-Fgr or NC. n = 6. C) Relative gene expression of Fgr in THP1-dMs transfected with Fgr overexpression plasmid or empty plasmid. n = 6. D) Fgr overexpression plasmid-transfected THP1-dMs were treated with DMSO or Api and colocalization of pHrodo red *E. coli* bioparticles (red), F-actin (green), and DAPI (blue) was shown. Scale bar: 10 μm. E) Phagocytosis of *E. coli* by THP1-dMs with Fgr overexpression or empty plasmid transfection for 48 h in the presence or absence of Api. n = 12. F-I) Western blot showing the expression of p-Vav1 (F), GTP-Rac1, GTP-Cdc42 (G), Arp2 and Arp3 (H), and quantification of results (I). n = 3 independent experiments. Data are presented as mean ± s.e.m. Statistical analysis was performed using two-tailed Student’s t-tests or One-way ANOVA with Sidak’s multiple comparison. *, *p* < 0.05. ns. not significant.
